# Age affects the immune system more than a moderate surgical trauma and anesthesia

**DOI:** 10.1038/s41598-025-26401-6

**Published:** 2025-11-07

**Authors:** Richard F. Kraus, Isabella Rastorfer, Sara Sixt, Tobias Hundhammer, Alexander Dejaco, Julia Rimboeck, Michael Gruber, Walter Petermichl

**Affiliations:** 1 Department of Anaesthesiology, University Hospital Regensburg, Franz-Josef‐Strauss‐Allee 11, 93053 Regensburg, Germany; 2 Department of Internal Medicine III, University Hospital Regensburg, Franz-Josef-Strauss-Allee 11 , 93053 Regensburg, Germany

**Keywords:** Neutrophil, T-cell, Age, Surface, Migration, Immune senescence, Immunological surveillance, Neutrophils, Acute inflammation

## Abstract

The effectiveness of the immune system decreases with increasing age. This process is known as immunosenescence. Recent studies showed the influence of aging on neutrophil granulocytes (PMNs) and T-cells, with the extent of the influence appearing to depend on various co-factors (such as the primary diseases of a patient). In this study, the PMNs and T-cells of younger and older adult patients were tested for their immunoreactivity before and after an operation in order to examine the consequences of the aging process on the moderately triggered immune system. Whole blood was taken from young patients (aged 18–65 years) and old patients (> 65 years) before and one day after an operation. Previous illnesses and medication intake were taken from the patient’s file. PMNs and T-cells were isolated. Immunoassays, live cell imaging (LCI) and flow cytometric examinations (FACS) were performed in order to assess certain properties of the PMNs (chemotactic migration, ROS production, NET formation, change of surface epitopes), their expression of adhesion molecules as well as their cell viability. In addition, the blood samples were subjected to a laboratory chemical examination. Above all during the initial LCI observation period (< 40 min), the PMNs of old patients covered longer distances than those of young patients. NETosis, ROS production and surface antigen expression were influenced neither by age nor by the surgical procedure. Regardless of age, PMNs´ ROS production started earlier 24 h after the operation compared to the pre OP values. By labeling the translocator protein (TSPO), it was demonstrated that mitochondrial release occurs only during suicidal NETosis. Old patients showed significantly more TSPO-labeled mitrochondria per PMN. The neutrophil-to-lymphocyte ratio (NLR) and the CD4/CD8 ratio were significantly increased in older patients. The share of CD28- and CD8-positive cells was increased in younger patients. All patients showed postoperative leukocytosis caused by an increase in monocytes and PMNs, which was independent of the extent of the trauma. Only young patients showed a postoperative increase in lymphocytes. Old patients had higher IL-6 levels than young patients. The operation did not lead to any increases in the IL-6 and CRP levels. Age influences the function of PMNs and T-cells more strongly than a moderate surgical trauma in combination with anaesthesia. The results advance our understanding of the decreasing effectiveness of the immune system in old age.

**Supplementary Information:**

The online version contains supplementary material available at 10.1038/s41598-025-26401-6.

## Introduction

### Immunosenescence

 Throughout their lives, humans are exposed to external influencing factors. Without corresponding defense mechanisms, pathogens (such as infectious agents) could spread unhindered in the body, and cause severe damage. However, the immune system is not only involved in the defense against pathogens, but also in other homeostatic processes in the body such as wound healing, regeneration, embryonic development, coagulation and tumor control^1,2^. Not only infections but also a surgical trauma or the tissue damage associated with it can trigger and influence immune responses. The extent of the influence depends on various co-factors such as primary diseases or the nutritional status of a patient. So far it is unclear which mechanisms are responsible for influences of the age on human immune reaction^3^.

**Fig. 1:**
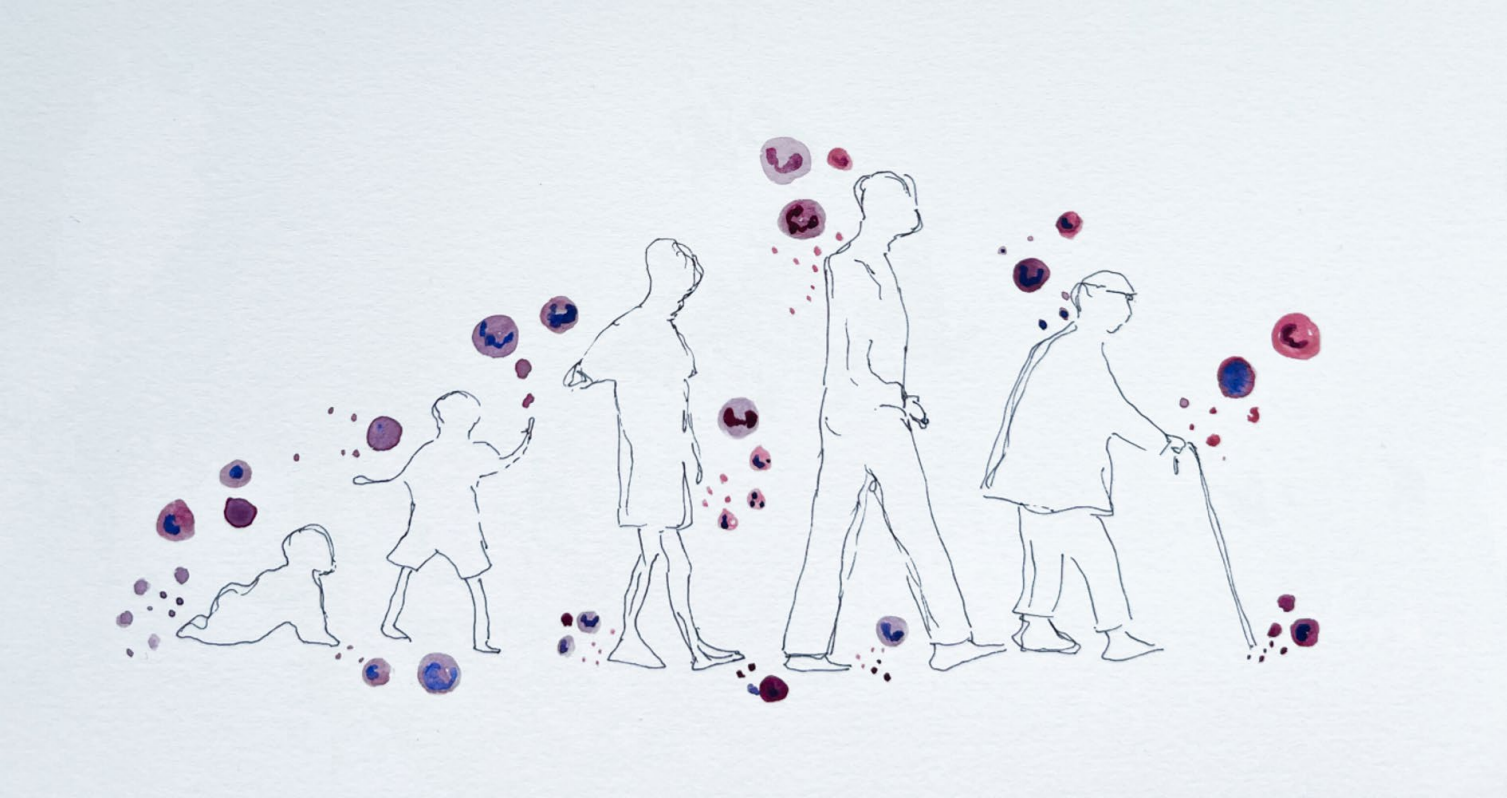
The effectiveness of the human immune system decreases with increasing age. This effect is known as immunosenescence. In our study, we demonstrated that age affects PMN and T cell function more than a moderate surgical trauma in combination with general anaesthesia.

The aging process results from the failure of evolutionary pressures to affect fitness at advanced ages^4^. Just like every other human organ, also the immune system is subject to an aging process (see Fig. 1). The term “immunosenescence” refers to a loss of effectiveness of the immune system in old age^5^. This age-related change in the immune system is one of the reasons for the increase in morbidity and mortality in old age^6^.

Signs of immunosenescence such as thymic involution or cell changes are found in many areas of the immune system^6^. The increasing life expectancy of humans, which is currently around 80 years, and the associated increase in diseases in the very old age group moves senescence studies into the spotlight of medical research^7^.

Seminal work has revealed important age-related differences in immune responses. Former research has largely centered on a narrow range of developmental and cell lineage markers. Furthermore, there are interactive and overlapping effects of sex and aging on the immune system. Newer studies profiled transcriptomes of peripheral immune cells sampled from women and men of two distinct age groups and revealed extensive differences in immune cell composition, molecular enriched pathways,, and cell-cell communication. Thereby, aging increases sex differences in circulating immune cells, particularly natural killer cells^8^.

Profiling of peripheral immune cells through single-cell RNA-sequencing coupled with single T cell and B cell receptor sequencing, high-throughput mass cytometry, bulk RNA-sequencing and flow cytometry validation experiments revealed, that the immune system undergoes dramatic changes during and after adolescence and sexual maturity. Thereby, T cells were the most strongly affected by age and experienced the most intensive reorganization of cell–cell interactions during specific age^9^.

The average human lifespan has increased in recent decades, which raises major health issues related to many age-associated pathologies. This research work aims to investigate and provide more insights into the influence of age on human immune reactions.

### Posttraumatic immunosuppression

Also sterile traumas (without tissue damage, without primary wound infection) trigger immunosuppression (the so-called posttraumatic immunosuppression, PTI)^3,10,11^. Depending on the extent of the trauma, the severity of immunosuppression varies from mild to severe. Also immunosenescence can increase PTI. PTI is a cause of postoperative infections or increased mortality. Both innate and acquired immune responses are influenced by a surgical or sterile trauma^3^. First, there is an increased chemokine and cytokine secretion (e.g. IL-6 and TNFα) followed by a systematic increase in neutrophil and monocyte counts due to their recruitment to the infection site. The inhibition of the hypothalamic-pituitary-adrenal axis results in an increased cortisol release in the course of the trauma. In addition, anti-inflammatory chemokines are released by endogenous tissue, the so-called “DAMPs”, to repair the trauma. A drop in natural killer cells and lymphocytes is also observed^10–12^. The exact process of immunosuppression and its prevention are not sufficiently researched yet^10,11^.

### Research objectives

**Fig. 2:**
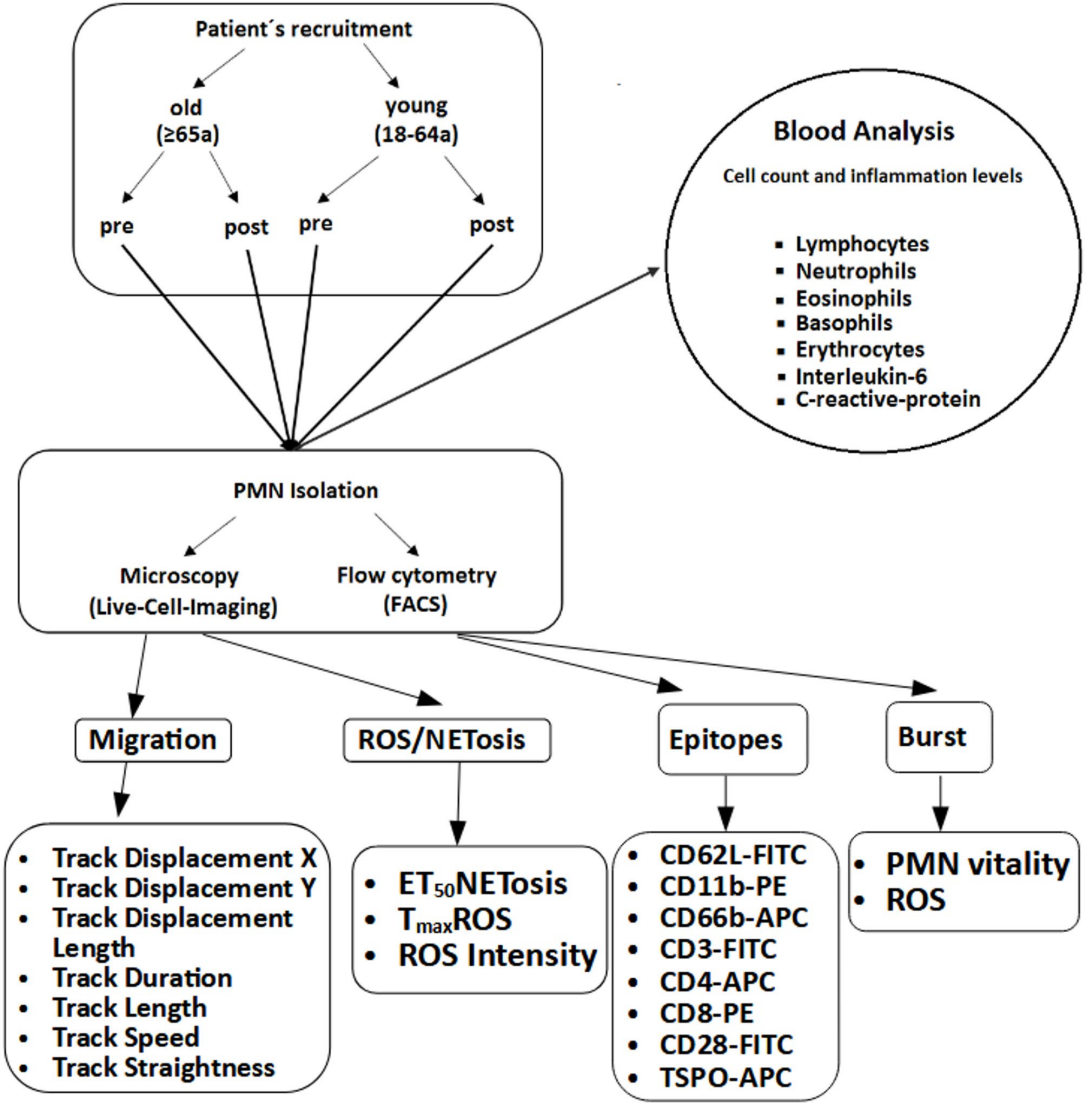
Study plan with the parameters acquired in the study.

The present research work investigated the immune response in old and young people after a defined stimulus (surgery) under general anesthesia. The behavior of PMNs was investigated by means of live-cell imaging with regard to their ROS production capability and their migration and NETosis behavior as a function of the patient´s age before and after an operation. Moreover, the release of mitochondria in PMNs during NETosis was examined by antibody-mediated staining of the translocator protein TSPO (18 kDa) (a receptor located at the outer mitochondrial membrane and involved in the regulation of cell proliferation, apoptosis, heme synthesis, mitochondrial steroid transport and steroid synthesis). In addition, T-cell and PMN surface epitopes were analyzed by means of flow cytometry (FACS). To be able to combine clinical laboratory parameters with research methods, live-cell imaging was matched to inflammatory response parameters (C-reactive protein (CRP) and interleukin-6 (IL-6)) and leukocyte counts. The study plan (see Fig. 2) visualizes all examined parameters and applied analysis methods.

## Materials and methods

For better reproducibility, a detailed list of materials can be found in the supplement in table S1.

### Vote of the ethics committee

The study was conducted in accordance with the principles of the Declaration of Helsinki and was approved by the local Ethics Committee of the Medical Faculty of the University of Regensburg (file number 19–1608-101).

### Patient´s blood recruitment

28 volunteer study participants were enrolled in this study from July 2020 to April 2021. The volunteers were patients who were in need of an eye or head-and-neck operation in general anesthesia with low tissue trauma and therefore expected low systemic inflammatory reaction during their inpatient stay at the University Hospital Regensburg. The patient´s recruitment was performed as follows: In distinct weeks during the above time period on Monday the surgery schedule for the following Tuesday was checked, and potential study participants were identified by means of age (> 18 years) and surgery. The patient who was scheduled as the 2nd point of the day was selected. Pregnant, anemic, or minor patients were excluded from the study. Also patients who had to undergo an emergency operation and patients with malignant tumors in their history were not included in this study. The patients´ participation was based on informed consent according to the Declaration of Helsinki. Patient-related demographic data (age, gender, height, weight) and health-related data (patient history, long-term medication) were taken from the patient file. All patient data were pseudonymized before the evaluation. The first blood sample was taken immediately before induction of anesthesia on the same day of the operation. The second blood sample was taken 24 h later. Lithium heparin monovettes of size 7.5 mL were used for both blood collections. Measurements on the day of surgery were titled “pre” and measurements on the day after were titled “post”. Patients were differentiated into “young” and “old”, “female” and “male”. Category “young” was defined from age 18 to 64 and “old” from age 65 and older.

### Blood count and inflammation values

On both test days, a blood count was obtained and the inflammatory values CRP and IL-6 were determined by the clinical chemistry laboratory at the University Hospital Regensburg.

### Cell isolation from patient’s blood

**Fig. 3:**
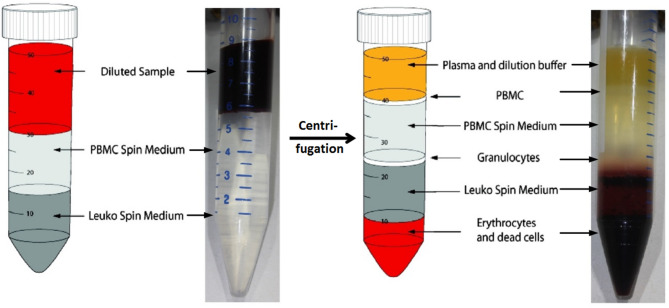
Schematic representation of density gradient centrifugation using the leuko/lymphospin technique.

The heparinized blood was divided for cell isolation and plasma collection. Leukocytes were collected by the density gradient centrifugation (leuko/lymphospin) as described before^13,14^. Afterwards, a typical phase separation into peripheral blood mononuclear cells (PBMC like lymphocytes) and granulocytes was recognizable in both tubes (see Fig. 3). After aspiration of plasma, 1 mL was removed from each of the two cell rings (lymphocytes and granulocytes).

(Graphic provided by PluriSelect Life Science UG & Co. KG: Density gradient media flyer (image modified by Richard Kraus). Available from URL: https://www.pluriselect.com/de_de/leuko-spin-medium.html#size=61 [Accessed 27 Oct 2023, 11:30 am].

### Plasma Preparation

For plasma preparation, 2 mL of patient whole blood was centrifuged at 385 g for 5 min.

### Live cell imaging

**Fig. 4:**
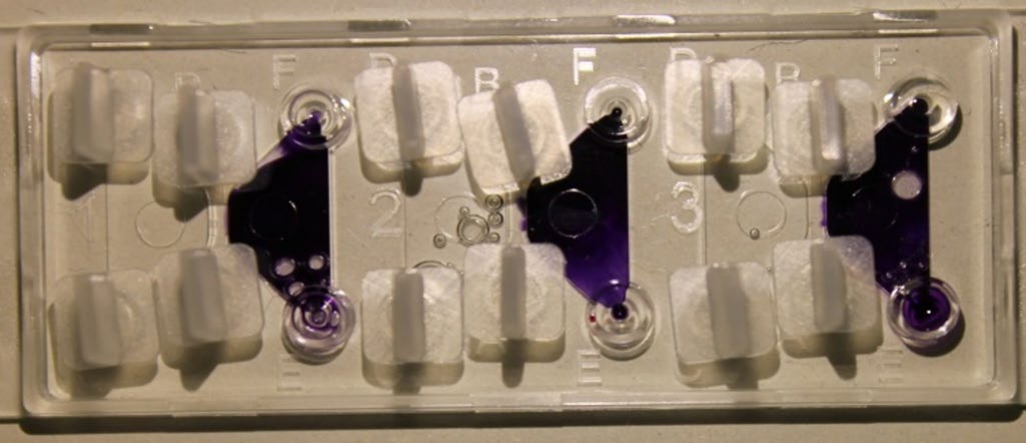
Composition of the IBIDI µ-Slide. For better illustration, the right reservoir was stained with blue hematoxylin solution in this figure.

Isolated granulocytes were applied at a concentration of 18 × 10^6^ cells/mL resuspended in autologous plasma for chemotaxis investigation with µ-Slide chemotaxis chambers^®^ (IBIDI GmbH). Briefly, these slides consisted of three channels that were filled with cells embedded in collagen gel (1.5 mg/mL PureCol, Advanced BioMatrix Inc., see Fig. 4)^15,16^.

Thereby, the left channel contained the gel with the dye-coupled antibody against MPO (Miltenyi Biotec, Bergisch Gladbach, Germany). The middle and right channels were filled with the gel containing the antibody against TSPO (SAB Biotech, C45244, College Park, MD, USA). All channels additionally contain DAPI (Sigma-Aldrich, Steinheim, Deutschland) and DHR (ThermoFisher Scientific, Eugene, USA). The slide was then incubated for 30 min in an incubator at 37 °C and 5% CO_2_ under humid conditions. After that, the reservoirs located on the left and right side of each channel were filled with plasma and RPMI 1640 as described before^13,17,18^. For all left reservoirs, the attractant fMLP was added in a final concentration of 10 nM to the cell culture media^13,17,18^.

**Fig. 5:**
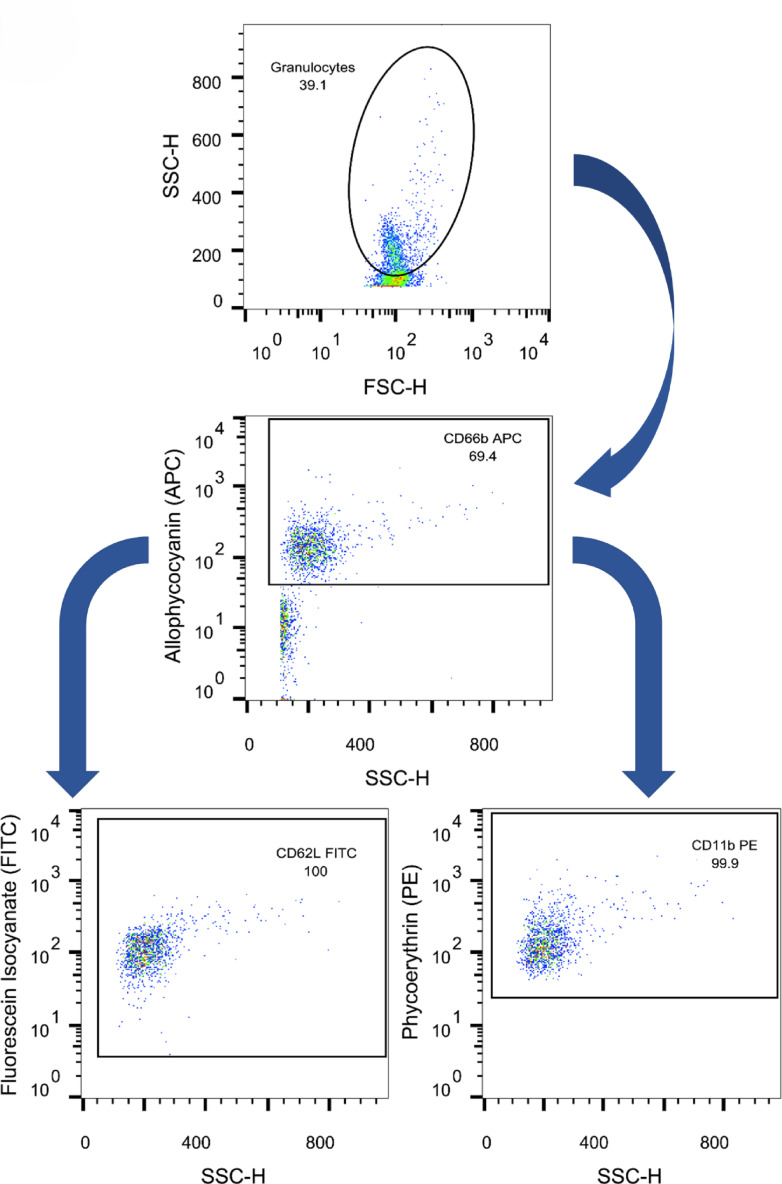
Examination sample of the expression of CD62L from isolated granulocytes using FITC conjugated antibody against CD62L. SSC-H = side scatter height FSC-H = forward scatter height

Neutrophil cell migration, ROS production, MPO release, NETosis and mitochondria ratio were documented using live cell imaging with a Leica microscope (DMi8, Leica Microsystems, Wetzlar, Germany) and an IBIDI Climate chamber (IBIDI, Martinsried, Germany) over a period of at least seven hours. Experiments were analyzed and scored for migration, ROS, MPO, NETosis and TSPO parameters using the Imaris program (Bitplane, Zurich, Switzerland). For migration analysis, separate time segments of 40 min each were evaluated, whereby the parameters listed in Table 1 were collected. To investigate the ratio of mitochondrial number to neutrophil cell count, the number of DAPI and TSPO areas was determined and compared at every 10th time point. The number of TSPO-bearing particles was related to the DAPI-bearing particles corresponding to the dimension of the neutrophil cell (see Fig. 5). The ratio of TSPO to DAPI dyed areas was taken as number of mitochondria per cell. Further data processing was performed by the external programs Excel (Microsoft, Redmond, USA), Phoenix (Certara, New York, USA), or SPSS (Statistical Package of Social Sciences, IBM Corporation, New York, USA) as describe before^13,14,19^.

**Table 1:** By live cell imaging observed parameters.

Parameter	Abbreviation	Unit
Track displacement length X	TDX	µm
Track displacement length Y	TDY	µm
Track length	TL	µm
Track speed max		µm/s
Track speed mean		µm/s
Track Speed Min		µm/s
Track straightness		-
Half-maximal time to maximal amount of DAPI stained cell	ET_50_NETosis	min
Half-maximal time to maximal amount of MPO stained cell	ET_50_MPO	min
Time to maximal ROS production	T_max_ROS	min
Ratio of mitochondrial number (TSPO) to cell count (DAPI)		-

### FACS

Parallel to the live cell imaging, FACS (Fluorescence Activated Cell Screening) analyses were performed. For this purpose, the already isolated PMNs and lymphocytes were used. For antigen expression analysis, antibodies were added to PMNs and lymphocytes. PMNs were stained with dye-coupled antibodies against CD11b, CD62L und CD66b (BioLegend, San Diego, each), lymphocytes were stained with antibodies against CD3, CD4, CD8 und CD28 (Invitrogen Life Technologies Corp., Carlsbad, each) as described before^13,18^. In addition, measurements with antibodies against TSPO were performed. Prior to TSPO measurement, a total of 1 mL ethanol was added to the isolated PMNs, followed by incubation for 30 min at room temperature. The ethanol was then removed by addition of 2 mL PBS, centrifugation for 5 min with 756 g and 4 °C. After removal of the supernatant, 50 µL PBS were added and cells were stained with 5 µL TSPO antibody (SAB Biotech, C45244).

**Fig. 6:**
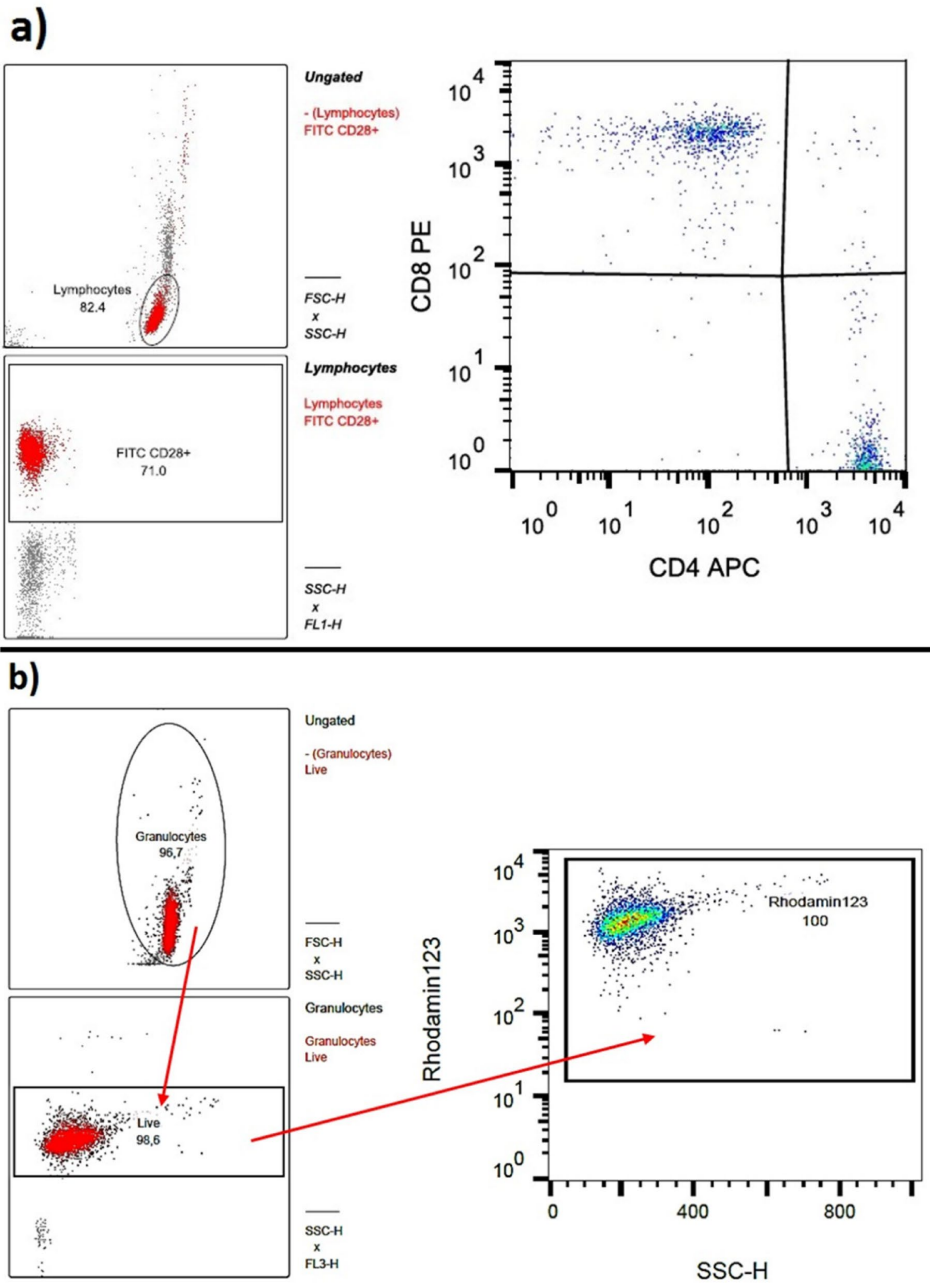
(**a**) Examination sample for the determination of the CD4/CD8 quotient (quadrant designation was clockwise Q1, Q2, Q3, Q4) (**b**) Examination sample FACS Burst. The sequence of the evaluation is shown with arrows

**Fig. 7:**
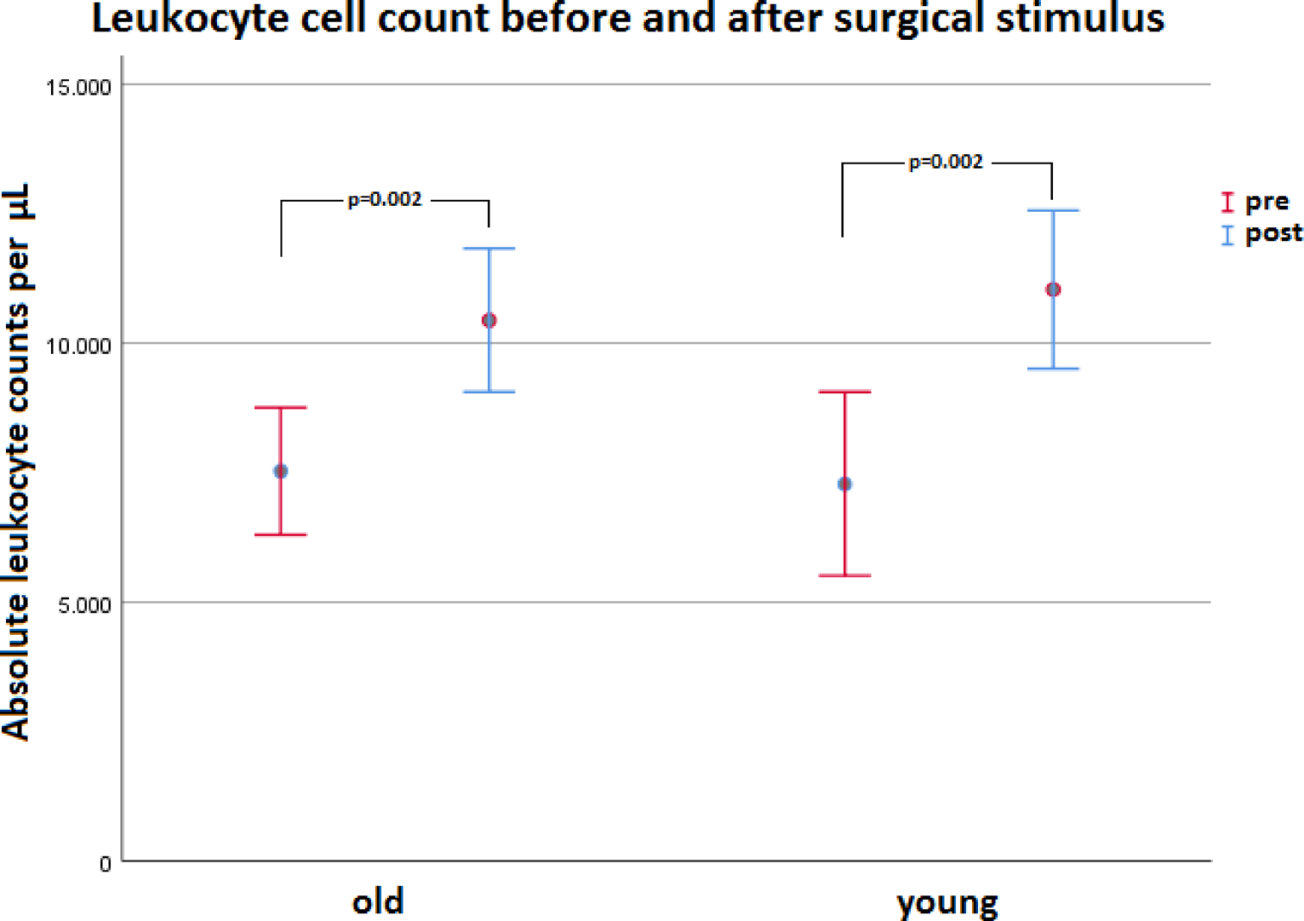
Results of absolute leukocyte cell count per µL of old (Mean_old_) and young (Mean_young_) patients before (red) and after (blue) the performed surgical intervention. Data are shown as mean ± 95% confidence interval.

Moreover, PMN ROS production was determined. The leuco dye DHR and SNARF were used for this purpose. Cells were primed with tumor necrosis factor alpha (TNFα) and activated with fMLP. PMA (phorbol 12-myristate 13-acetate) served as the activator for the positive control. PMA was used without TNFα. PMNs were first mixed with DHR, SNARF, and TNFα and incubated. In the second step, PMA and the activating agent fMLP were added. After re-incubation, the dye propidium iodide (PI) was used to label the dead cells. All measurements were performed using the Cellquest Pro program (Becton Dickinson)^18^. The data were analyzed with FlowJo (V10.0.7 FlowJo, LLC, Ashton, Oregon, USA, see Figs. 6 and 7) and then further processed with Excel and SPSS as described by Doblinger et al.^13^.

### Statistical analysis

The significance level (p-value) was set at 0.05 for all tests. The Kolmogorov-Smirnov test was used to test the data for normal distribution. In the case of normal distribution (*p* > 0.05), the t-test was performed for two variables, observing two-sided significance for independent or connected samples. In case of more than two variables, one-way analysis of variance (ANOVA) was performed. Data are presented as scatter plots with mean and standard deviation (95% confidence interval). Prior to the application of Kruskal-Wallis and ANOVA testing, analysis for variance homogeneity was performed using Levene’s tests. Post-hoc analysis according to Bonferroni (for variance homogeneity) or Dunett-T3 (for variance inhomogeneity) was performed accordingly. For non-normally distributed data, the non-parametric Mann-Whitney U test was used for two variables or the Kruskal-Wallis test with pairwise comparisons for more than two variables. For some analyses, we additionally computed statistical power (power). Results are presented in the form of a simple or grouped boxplot. In all figures statistical outliers are represented as circles, and extreme values are depicted as asterisks.

## Results

A total of 28 patients were examined. Their demographic data are displayed in Table 2 and in more detail in Table S2 in the supplement.

**Table 2:** Demographic data of the participants.

Age group	18–64 J (young) Average age ± SD [years] (number)	> 64 J (alt) Average age ± SD [years] (number)
Female	33.4 ± 7.3 (5)	82 ± 6.7 (5)
Male	28.8 ± 4.6 (5)	78.9 ± 3.8 (13)
Patients (total)	31.1 ± 6.5 (10)	79.9 ± 5.0 (18)

### Leukocytes

**Fig. 8:**
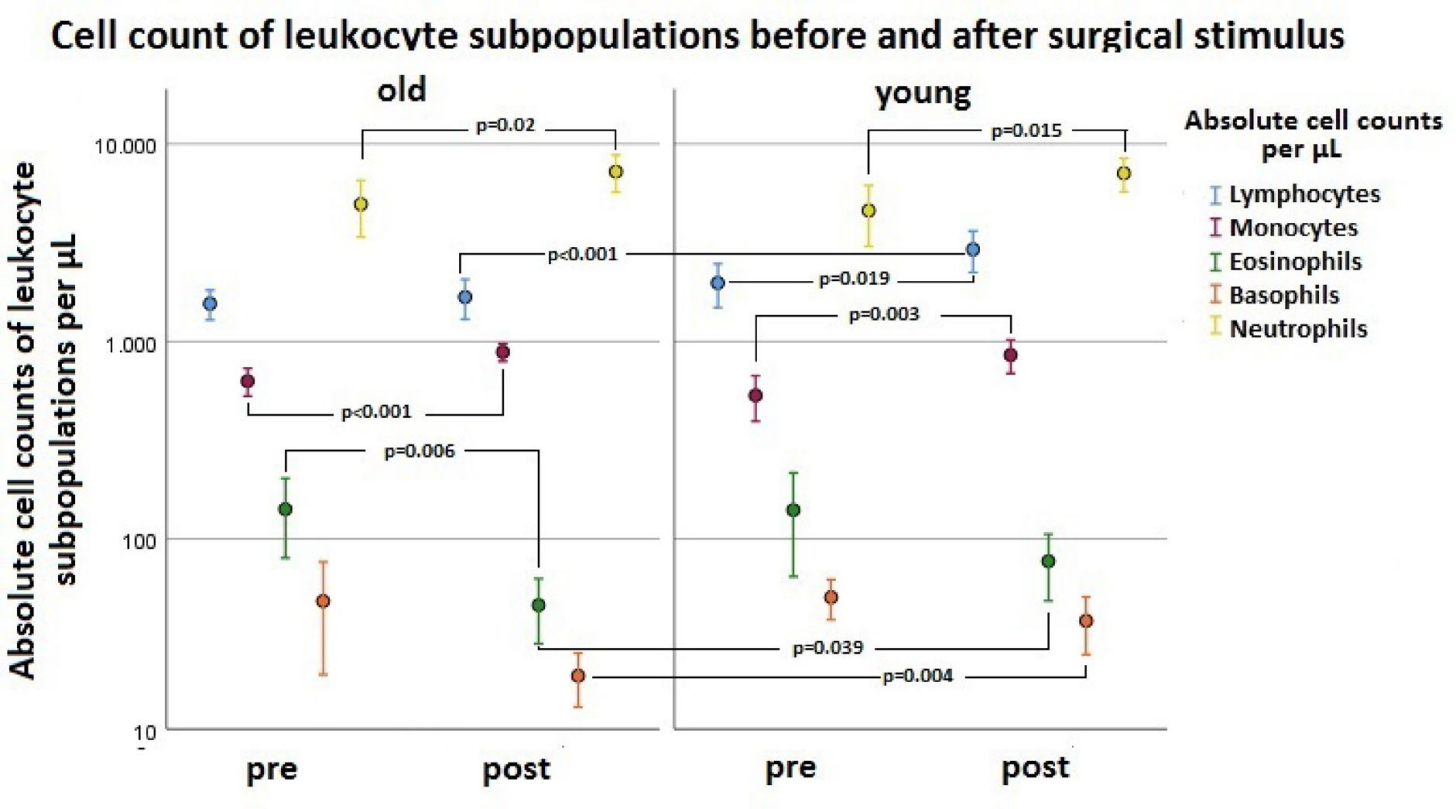
Results of absolute cell counts of leukocyte subpopulations of old (Mean_old_) and young (Mean_young_) patients before and after surgical stimulus: lymphocytes (blue), monocytes (red), eosinophils (green), basophils (orange) and neutrophils (yellow).

A comparable significant increase in absolute leukocyte count from pre (Mean_old_ = 7529 ± 2466 abs/µL, Mean_young_ = 7283 ± 2477 abs/µL) to post (Mean_old_ = 10439 ± 2787 abs/µL, Mean_young_ = 11034 ± 1991 abs/µL) was found in both old and young patients (p_old_ = 0.002, p_young_ = 0.002; see Fig. 8).

### Subgroup analysis of leukocyte populations

**Fig. 9:**
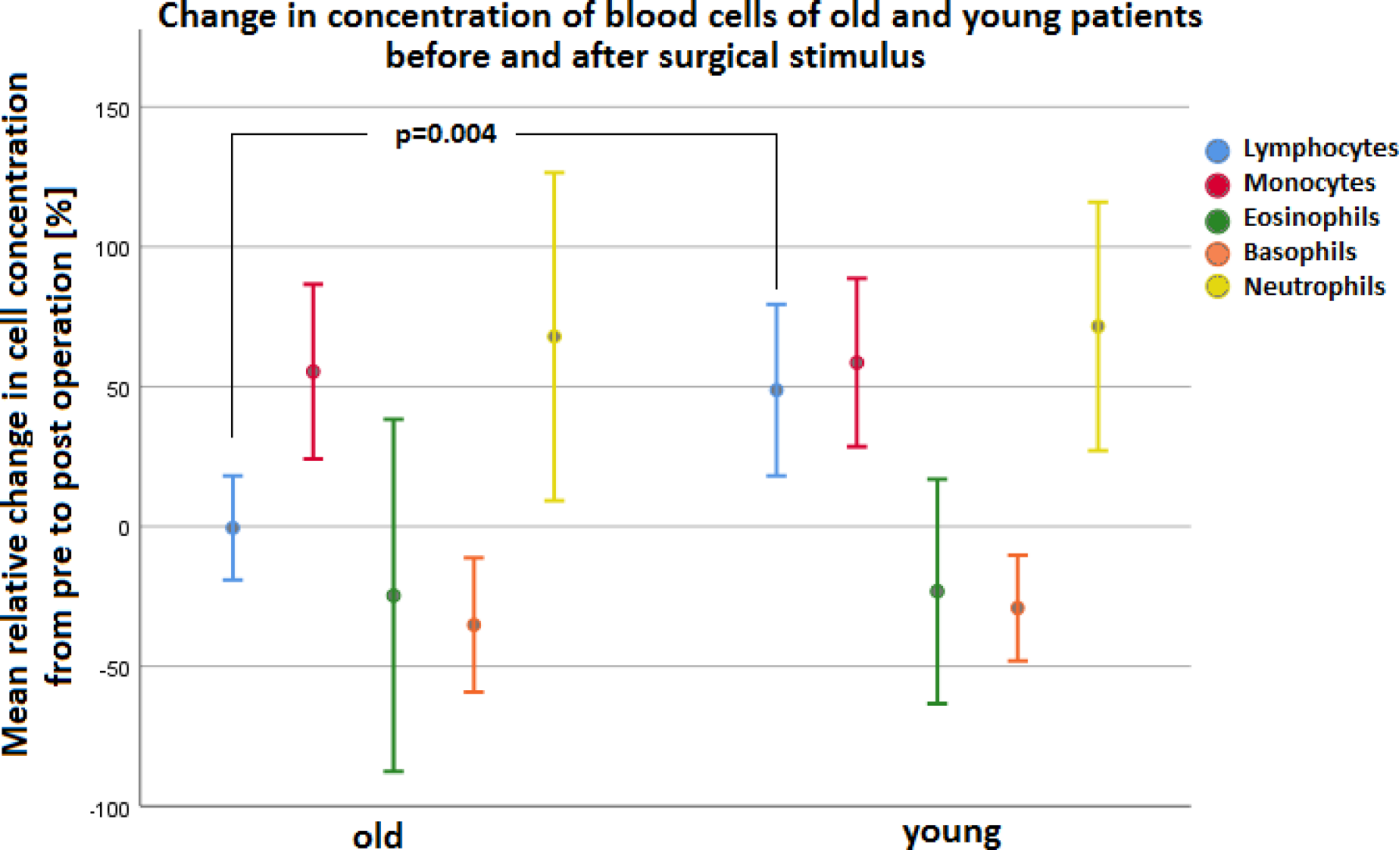
Results of the mean relative change in concentration (Mean_old_ /Mean_young_ in %) of all blood cells in old and young patients before and after surgical stimulus: lymphocytes (blue), monocytes (red), eosinophils (green), basophils (orange), neutrophils (yellow) and erythrocytes (turquoise). Mean statistical differences between the individual age groups are shown only in the lymphocyte difference (turquoise).

The divided analysis of the leukocyte subgroups manly showed an increase of monocytes and neutrophils for elderly people from pre to post. In contrast, basophil and eosinophil granulocytes decreased in percentage from pre to post in both old and young participants. Only the lymphocyte percentage changes significantly depending on the age group, whereby for young patients additionally the lymphocytes ascended (see Fig. 9 and Table S3 in the supplement).

**Fig. 10:**
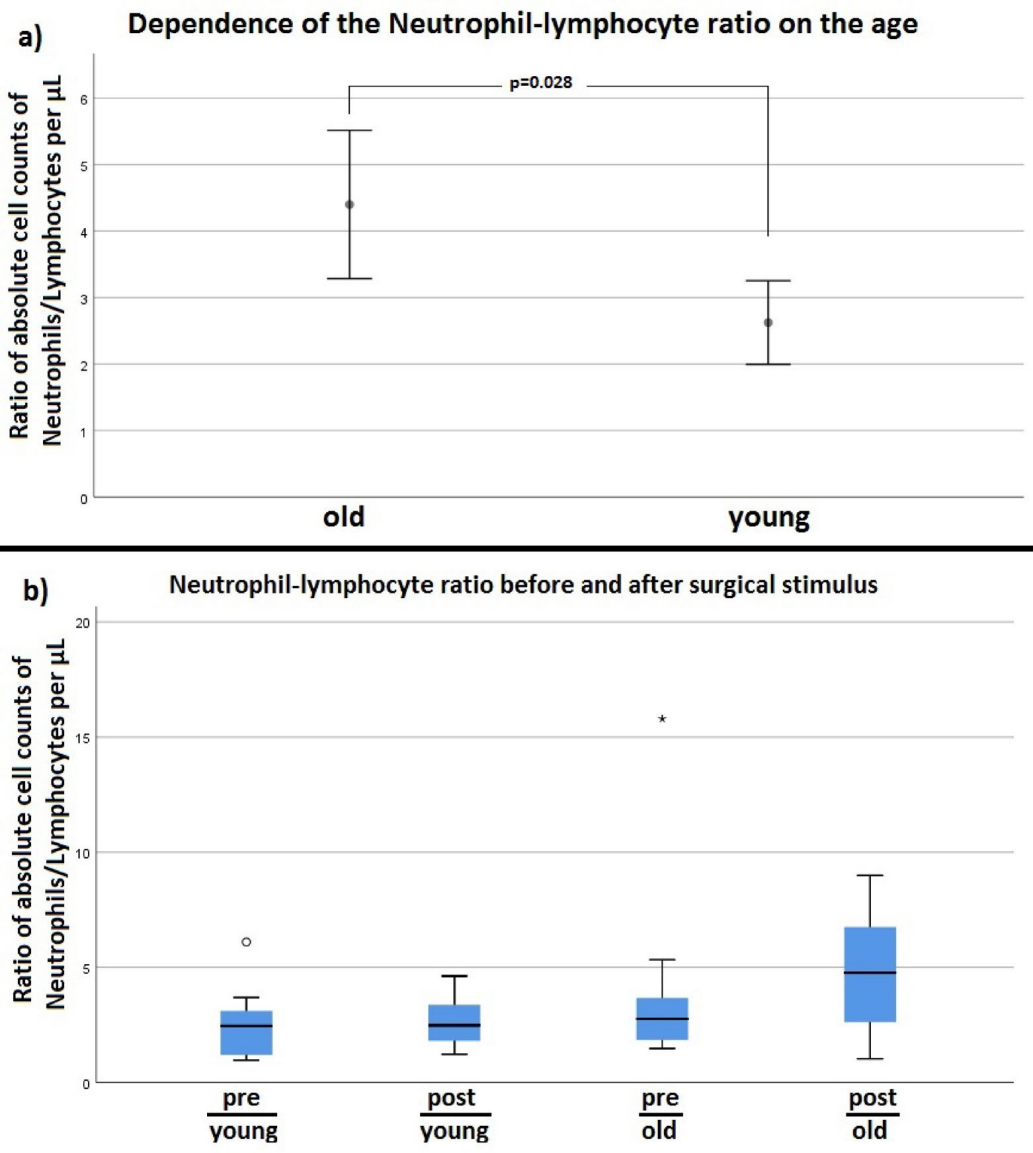
(**a**) Neutrophil-Lymphocyte-Ratio in old (Mean_old_) and young (Mean_young_) patients without differentiation in pre- or postoperative (**b**) Neutrophil-Lymphocyte-Ratio before and after surgical stimulus divided into the various combination classes pre/young, post/young, pre/old and post/old. (pre=preoperative, post=postoperative, old= (Median_old_ young =Median_young_ )

The absolute leukocyte counts of the subgroups were compared before and after surgery with the total leukocyte counts. Only the lymphocyte concentration changed in dependence of the age group (see Fig. 10 and Table S3 in the supplement).

### Neutrophil-lymphocyte ratio

**Fig. 11:**
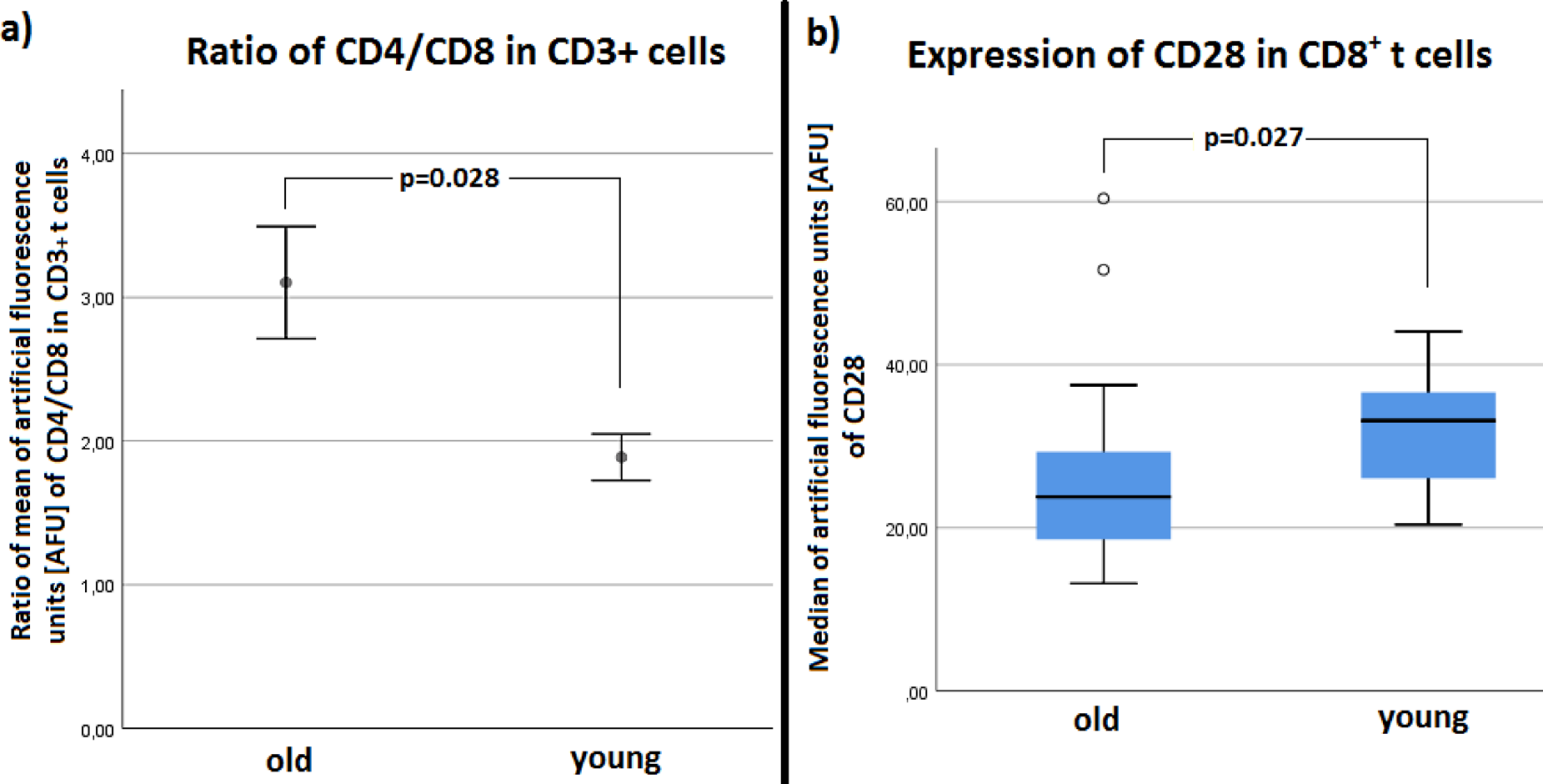
(**a**) Comparison of Mean_young_ and Mean_iold_of CD4/CD8 ratio of CD3+ T cells of young and old patients. Old patients showed a higher CD4/CD8 ratio than young patients (**b**) FACS analysis showed a significant higher Median_young_AFU of CD28 in CD8^+^ T cells

Without distinction whether before or after surgery young participants overall had a significant higher neutrophil-lymphocyte ratio (Mean_young_ = 2.482) than old participants (Mean_old_ = 3.603) with *p* = 0.028 (power = 0.76) (see Fig. 11a). Direct comparison of the individual groups with each other showed a significant difference (*p* = 0.043, power = 0.82) by Kruskal-Wallis test. However, there was no value of the adjusted significance < 0.05 (Dunn-Bonferoni) (see Fig. 11b and Table S4 in the supplement).

### C-reactive protein and interleukin-6

IL-6 concentration increased from pre (Median_old_ = 3.400 pg/mL) to post (Median_old_ = 4.800 pg/mL) in old patients. In young patients, IL-6 concentration also increased from pre (Median_young_ = 2.500 pg/mL) to post (Median_young_ = 2.950 pg/mL). The CRP concentration of old patients increased from pre (Median_old_ = 0.800 mg/L) to post (Median_old_ = 1.500 mg/L). In young patients, this also increased from pre (Median_young_ = 0.800 mg/L) to post (Median_young_ = 0.950 mg/L). However, the Mann-Whitney-U test did not show a statically significant change from preoperative to postoperative for both age groups for IL-6 and CRP. Without division into pre and post, a significant difference between young and old patients was found for IL-6 (*p* = 0.006, power = 0.53) but not for CRP (*p* = 0.260, power = 0.75).

### Detection of CD11b, CD62L, CD66b and TSPO on/in PMNs and ROS

A decrease in AFU values was found for all antigens in both age groups from pre to post, except for CD66b in young patients (Exact values are displayed in Table S5 in the supplement). None of these decreases reached statistic significance. The Medians of ROS detection (DHR) with FACS showed no significant differences for ROS production and the oxidative burst, neither for fMLP nor for PMA stimulation (exact results are reported in Table S6 in the supplement). TSPO measurements by flow cytometry (FACS) were not performed, so there are no TSPO intensities reported from FACS.

### Lymphocyte ratios

**Fig. 12:**
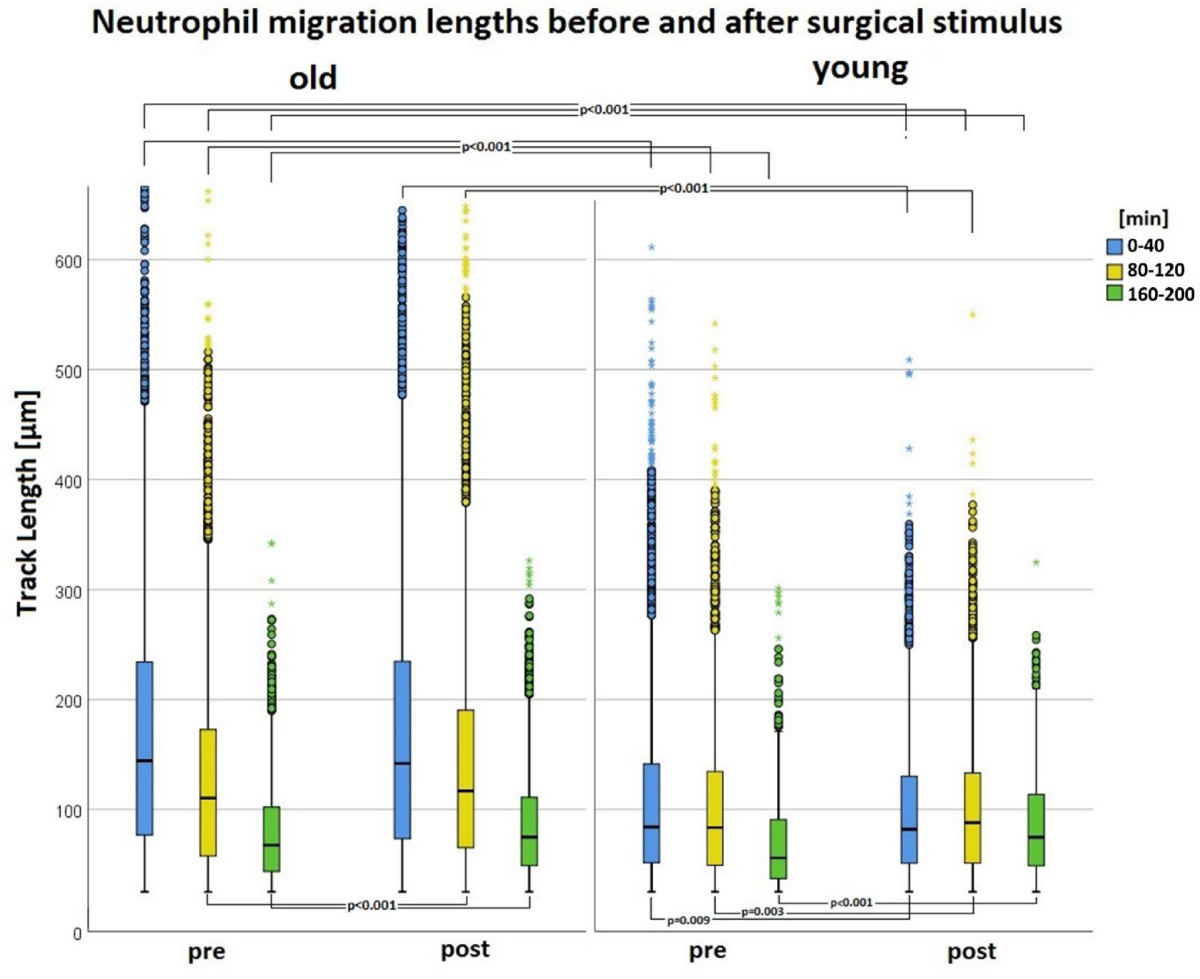
Comparison of neutrophil migration lengths between old and young patients pre and postoperative at time periods 0-40 min (blue), 80-120 min (yellow), and 160-200 min (green). The migration lengths decreased with increasing observation time. There were statistically significant differences between the migration lengths of young and old patients (see marked p‑values).

In the single-group analysis (*p* = 0.12, power = 0.64), a significant influence of the surgical stimulus was excluded, so no distinction was made between pre- and postoperative. Old patients had a significant higher CD4/CD8 ratio (Mean_old_ = 3.10) than young patients (Mean_young_ = 1.89) with *p* = 0.028 (power = 0.76) (see Fig. 12a). However, young patients (Median_young_ = 33.1) showed a significant higher expression of CD28^+^ CD8^+^ than old patients (Median_old_ = 23.8) with *p* = 0.027 (power = 0.76) (see Fig. 12b).

## Results of live-cell-imaging

### Parameter migration

**Fig. 13:**
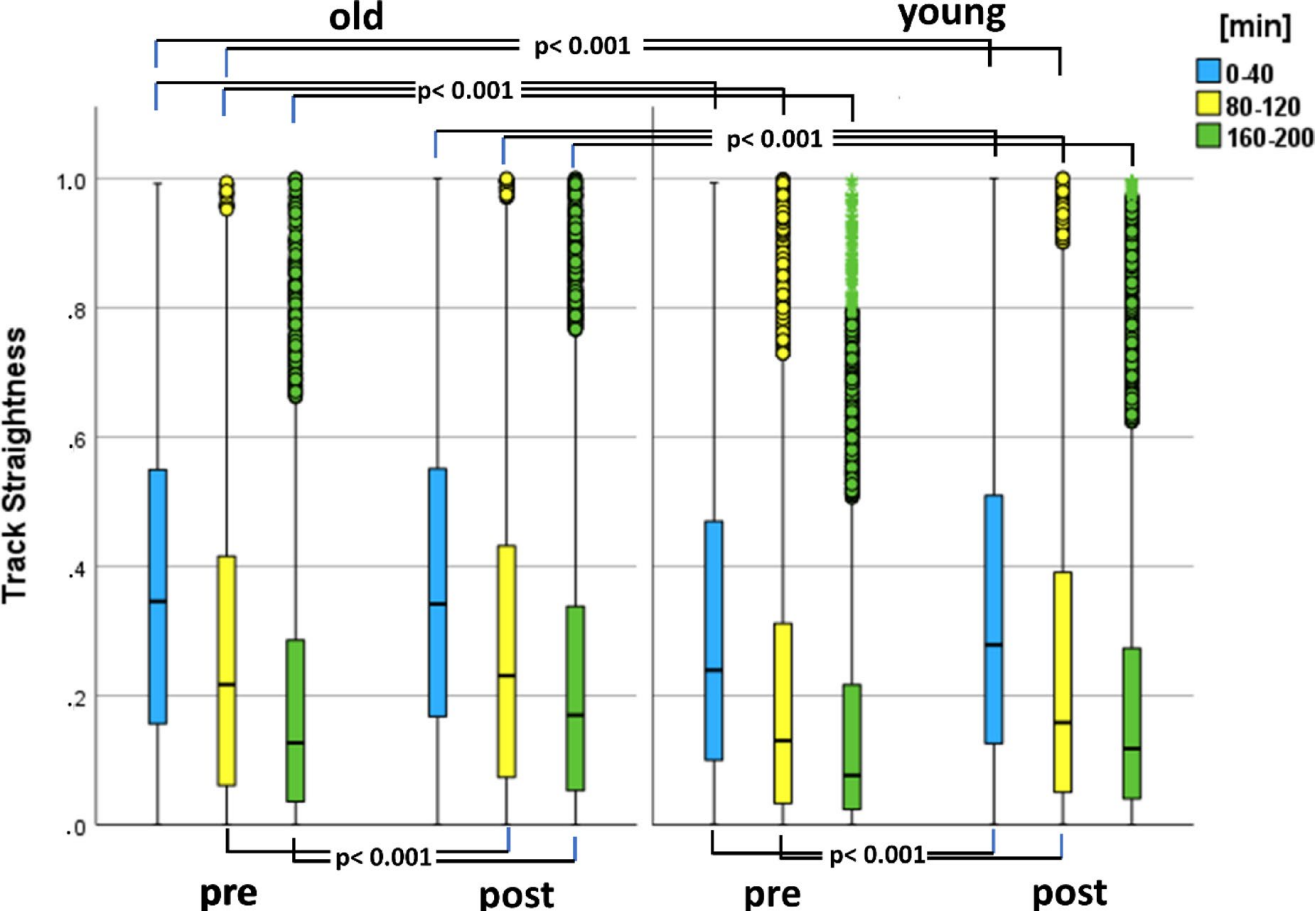
Comparison of neutrophil directional accuracy (straightness) between old and young patients pre and postoperative at time periods 0-40 min (blue), 80-120 min (yellow), and 160-200 min (green). The straightness decreased with increasing observation time. There were statistically significant differences between the straightness of young and old patients (see marked p‑values).

PMNs of old patients covered greater distances than those of young patients both preoperative and postoperative. Preoperative Median_old_(TL) was in all time periods (with exception of 160–200 min) significant higher than Median_young_(TL) (see Fig. 13; *p* < 0.001). Median_old_(TL) was postperative higher than preoperativ (*p* < 0.001; except for time period 0–40 min). Median_young_(TL) also was postoperative higher than preoperativ (p values see Fig. 13). Even preoperative Median_old_(TL) was higher than postoperative Median_young_(TL) (*p* < 0.001). Our results showed that in all analysis groups, the determined track lengths diminished with increasing duration of the experiment (see Fig. 13; *p* < 0.001). At every point of measurement, the distance of TDY was less than of TDX.

**Fig. 14:**
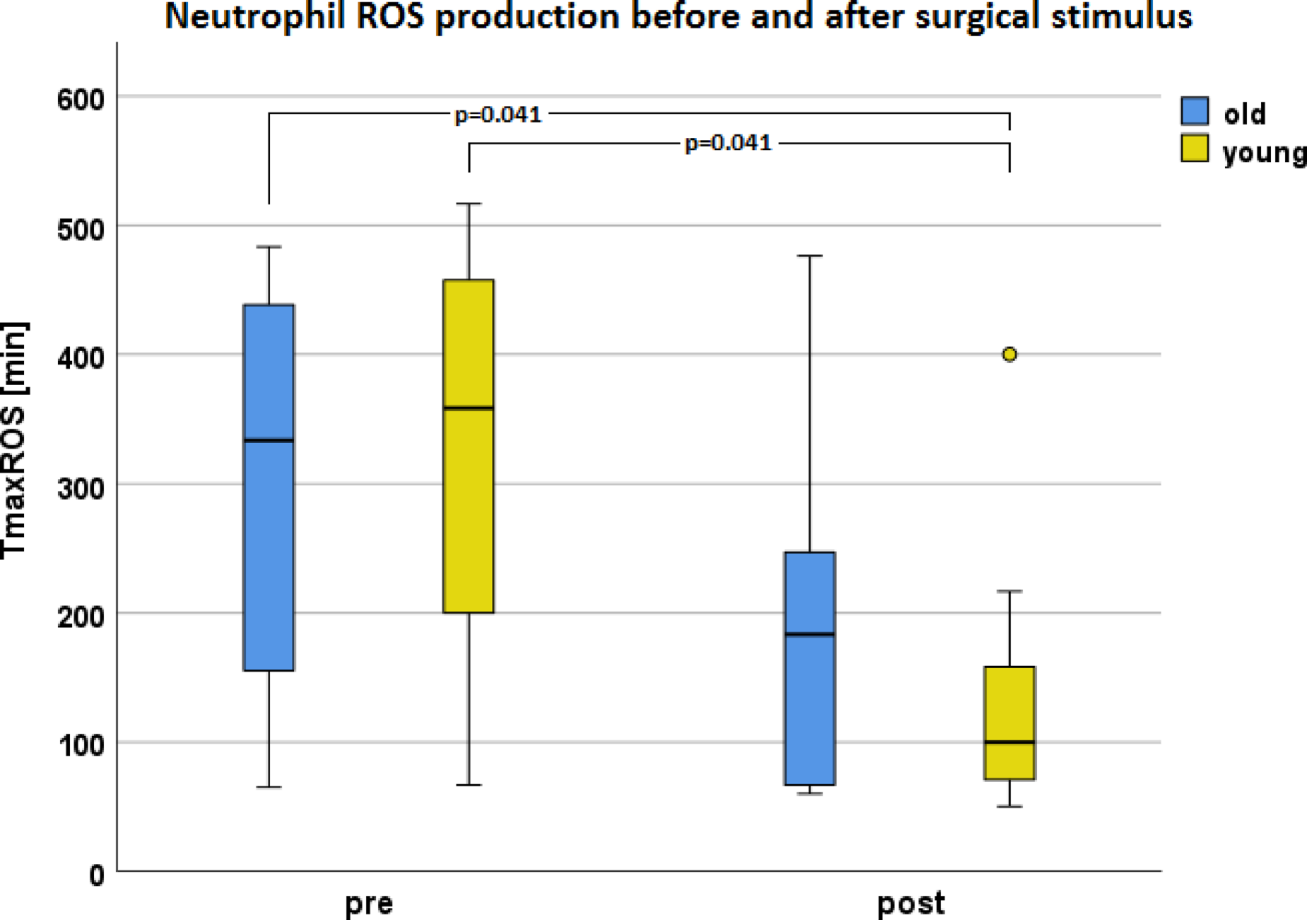
Comparison of TmaxROS [min] pre- and postoperatively depending on age.

In addition, PMNs of old patients showed greater directional accuracy (straightness) than those of young patients both preoperative and postoperative. Preoperative Median_old_(TS) was in all time periods significant higher than Median_young_(TS) (see Fig. 14; *p* < 0.001). Median_old_(TS) was postperative higher than preoperativ (*p* < 0.001; except for time period 0–40 min). Median_young_(TS) also was postoperative higher than preoperativ (except for 160–200 min). Even preoperative Median_old_(TS) was higher than postoperative Median_young_(TS) (*p* < 0.001).

### ROS production – live cell imaging

**Fig. 15:**
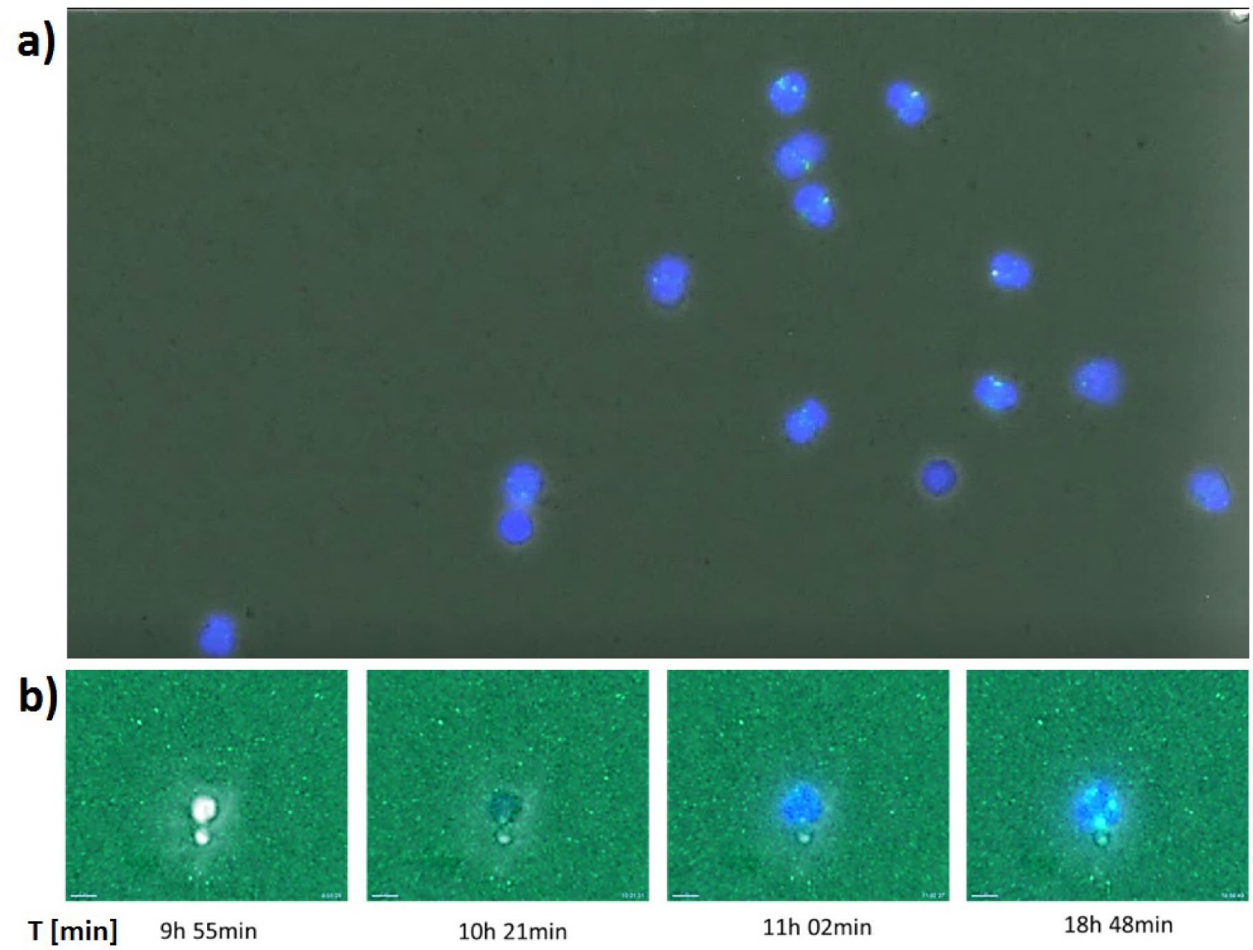
Post-NETotic staining of the TSPO protein in the outer mitochondrial membrane. (**a**) Illustration of the TSPO (green) to DAPI (blue) areas. After a neutrophil cell performs NETosis (visualized by DAPI (blue) using DNA staining), TSPO (green) is located in the mitochondria of the neutrophil cell, whereby TSPO is visualized by anti-TSPO antibodies. (**b**) Time flow [min] of a neutrophil cell treated with fMLP showing suicidal NETosis (blue, DAPI, staining PMN–DNA (NET)). TSPO (green) is located in the mitochondria walls of neutrophils and is visualized by anti-TSPO antibodies. The number of TSPO areas (green) correspond to the number of mitochondria and the DAPI areas to the cell number. The length of the superimposed bar (bottom left) corresponds to 7 µm. The number of DAPI and TSPO areas was determined. In simple terms, the TSPO areas correspond to the number of mitochondria and the number of DAPI areas mirror the number of cells. The number of TSPO-bearing particles was related to the DAPI-bearing particles. The ratio of TSPO to DAPI dyed areas was taken as mitochondria per cell.

Parameter T_max_ROS showed postoperative an earlyer Median_young_ (100.0 min [IQR 158.3 min]) than preoperative Median_young_ (358,3 min [IQR 337,0 min]) and preoperative Median_old_ (333,3 min [IQR 333,2 min]) (see Fig. 15; *p* = 0.041). Without distribution to the age groups, there was a significant change of T_max_ROS from preoperative (Median_pre_ = 358.3 min) to postoperative (Median_post_ = 100.0 min; *p* = 0.020, power = 0.72). In various channels Tmax calculation was not possible due to an undefinable maximum or not visible ROS production in the Imaris evaluation (Table S7 in the supplement shows the number of channels which could be evaluated).

### ET_50_NETosis and ET_50_MPO

No significant difference was found between ET_50_NETosis values (see Table S8 in the supplement) in old and young patients pre- and postoperative (*p* = 0.766). The ET_50_MPO values (see Table S8 in the supplement) also showed no significant difference between the individual groups (*p* = 0.685).

### Ratio of mitochondrial number to cell count

The process of determining the ratio of mitochondrial number to cell count is visualized in Fig. 5. The ratio of mitochondrial number to cell count significantly differed from old (Median_old_ = 1.571) to young patients (Median_young_ = 0.716) with *p* = 0.001.

The number of DAPI and TSPO areas was determined. In simple terms, the TSPO areas correspond to the number of mitochondria and the number of DAPI areas mirror the number of cells. The number of TSPO-bearing particles was related to the DAPI-bearing particles. The ratio of TSPO to DAPI dyed areas was taken as mitochondria per cell.

## Discussion

This study investigated the impact of a stress stimulus (in this case a surgical intervention) on functions of neutrophil granulocytes and T cells in adult patients under and over 65 years of age.

Both patient groups showed postoperative leukocytosis caused by an increase in monocytes and PMNs (see Figs. 8 and 9). The total lymphocyte count only of young patients was increased after the operation (see Fig. 9). Without differentiation whether before or after surgery, old patients overall showed a higher neutrophil-to-lymphocyte ratio (NLR) (see Fig. 11a). However, after differentiation in young and old patients and before and after intervention NLR did not reach significance in the groups, respectively. Consequently, young and old patients did not differ significantly in the NLR from pre- to postoperative. The described difference in NLR of young and old patients in our study (see Fig. 11a) was mainly due to post/old measurements (see Fig. 11b). However, Fest et al. and Li et al. described recently, that NLR is higher in higher age categories^20,21^ and it is evident that an increased NLR in the postoperative period after hip surgery is associated with an increased mortality^22,23^.

The CD4+/CD8+ ratio was significantly increased in old patients. The proportion of CD28+ in CD8+ cells was increased in younger patients independently of the pre/post status. Old patients had higher IL-6 levels than young patients. The operation did not lead to any increase in IL-6 and CRP. The neutrophils of old patients covered longer distances than those of young patients. MPO release, NETosis and surface antigen expression were influenced neither by age nor by the operation. Regardless of age, ROS production started earlier after the operation. Old patients showed significantly more TSPO-labeled mitochondria per PMN cell.

### Selection of patient groups

For this study, patients from 65 years onwards were selected for the “old” age group as this age appears to be associated with an increase in dysregulation. According to the classification of the “Silver Books” of the Alliance for Aging Research, chronic diseases such as hypertension, arthritis, coronary diseases, diabetes, or cancer increasingly appear from the age of 65 years onwards. 80% in this age group had one or more of these chronic diseases^24^. Thus, Patients over 64 years increasingly suffer from type 2 diabetes^25^. Moreover, a study by Campisi J. (2013) showed, that cell aging begins earlier, namely from 50 to 60 years onwards. Senescent cells have, for example, a lack of tumor suppression, or promote degenerative aging-related diseases^26^. It is unclear if a different classification into “old” and “young” had led to different results. But beware, chronological age is not always equal to biological age.

### Changes in cell counts and blood laboratory values in young and old patients

This study showed an increase in the total leukocyte count in both test groups after a surgical stress stimulus. Similar to our study, Jung et al. examined patients undergoing distal pancreatectomy showing a leukocyte number reaction. For classification, a division into a group with high leukocyte count (> 20,000 cells/µL) and a group with low leukocyte count (< 20,000 cells/µL) was made there. Compared to the values before the operation, a strong increase in the leukocyte values of both groups could be observed on the first day after the operation. A leukocyte count of > 20,000 cells/µL could be observed in only 4.6% of the study participants, which was associated with an additionally performed splenectomy. High leukocytosis was often an indicator of an inflammation or an infectious complication, but was never associated with a surgical complication^27^. The study conducted here also showed an increase from before the operation to the first day after the operation which was comparable to that of the < 20,000 cells/µL group. In the study conducted here, however, a smaller surgical intervention compared to the minimally invasive pancreatectomy was performed, which may have resulted in lower tissue stress. Presumably, the leukocyte count in the study conducted here was thus reduced in comparison. Anyway, the leukocyte count with postoperative Mean(leucocyte count) = 12,270 cells/µL was comparable to our values with Mean(leucocyte count) = 10,737 cells/µL. From the data collected in the studies it may be deduced that leukocyte liberation is independent of trauma severity. Simply speaking, at least the early leukocytosis reaction until day 2 (without additive infection) appears to work according to the all or nothing principle. Leukocytosis can also be a reaction to acute stress. An animal test study by Dhabhar et al. showed leukocytosis followed by a steady decrease in the total leukocyte count as early as 6 min after rats were exposed to an acute stress stimulus^28^. Hence, leukocytosis is a nonspecific stress response and does not necessarily have to be caused by surgical stress alone.

In order to gain a more detailed insight into the cause of the leukocyte increase, the individual leukocyte subpopulations were examined: neutrophil granulocytes, monocytes, lymphocytes eosinophil and basophil granulocytes. Both in old and young patients, the postoperative leukocyte increase in percentage terms was above all due to an increase in monocytes and neutrophil granulocytes. This was also confirmed by a look at the absolute figures of the individual measurement values. The same was already found in other studies. Richter, Hans Peter showed an increase in the absolute and relative content/µL of neutrophils and monocytes as early as 6 h after cardial bypass surgery, which persisted up to 48 h after the operation. However, the patients had an average age of 65 years^29^. Another study by Sbrana et al. also described a postoperative increase in leukocytes in the coronary blood, especially an increase in granulocytes and monocytes, after cardiac surgery (aortic valve replacement with or without bypass) with cardioplegia. This increase was seen both 5 h and 24 h after the operation performed. 20 patients aged 43 to 85 years were examined here^30^.

In both age groups, the cell concentrations of basophil and eosinophil granulocytes dropped in percentage terms after the operation. However, when looking at the absolute figures, a significant drop in the cell count of eosinophils and basophils was observed only in old patients. Young patients showed no statistically significant change. In the above-mentioned study of Richter, Hans Peter, the absolute numbers/µL and percentages of total lymphocytes were significantly decreased immediately after anesthetic induction for up to 48 h after the operation. The same was observed for the course of the absolute eosinophil numbers. Here, too, there was a clear drop in the absolute count after the operation^29^. Comparable results were described in a study by Miller et al. Here, too, there was an increase in PMNs after the operation. At the same time, there was a drop in eosinophil granulocytes and lymphocytes^31^.

The peripheral lymphocyte drop after surgery (especially in young patients) represents a surgery-induced immunosuppression caused by effects on the cellular components of the immune system. Possible reasons are that circulating lymphocyte numbers fall peri-operatively, possibly due to a decrease in the lymphocyte proliferation rate or a redistribution of lymphocytes from the peripheral blood to the body compartments^32^. Nevertheless, peri-operative stress can lead to an increase in endogenous cortisol release (young > old), which promotes lymphocyte apoptosis and redistribution of lymphocytes to tissues. Exogenous corticosteroid exposure, such as perioperative steroids (for example dexamethasone as prophylaxis for postoperative nausea and vomiting (PONV)), can transiently suppress lymphocyte counts, whereas neutrophils and monocytes may be less affected or even increased^33^. However, in our study the exogenous cortisol supply was administered to both older and younger patients. This might preclude the iatrogene application of dexamethasone alone as the sole explanatory factor for these observed effects.

Although a bypass operation in general anaesthesia or cervical cancer surgery represents a considerably more severe tissue trauma and the age classification differs, the results obtained in our study are comparable to the existing literature^31,32^. By determining the differences of the measurement pairs for each individual patient, also young patients show a clear tendency for the values to drop even though the drop in eosinophil granulocytes could not be proven statistically. The percentage decrease can also be due to an overexpression of the other cell lines as the relative percent value of eosinophil granulocytes appears lower as a result.

From these findings it can be deduced that an increase in monocytes and neutrophils occurs in response to a trauma regardless of its severity above an unevaluated threshold. A drop in eosinophils and basophils also occurs as a consequence regardless of the severity of the trauma. This reaction presumably happens according to the all-or-nothing principle as the body does not differentiate here between severe or mild tissue damage.

In our study there was no significant change in the absolute lymphocyte counts in old patients from before to after the operation. In young patients, an increase in lymphocyte count was observed postoperative. A recent study with subjects with an average age of 66 years showed a decrease in CD4+ and CD8+ lymphocytes and an increase in all blood leukocytes two days after the operation^34^. However, in the study conducted here, blood sampling was done on the first day after the operation and also showed an increase in all blood leukocytes. The total lymphocyte count (B and T lymphocytes) in old patients with an average age of 79.9 years was unchanged, which could be due to the fact that not only T lymphocytes were monitored here. In 1984 Hamid et al. showed that the total lymphocyte count drops by up to one third of the initial value (pre) two days after an operation. This drop could be detected from a period of 2 h after the operation performed. Besides, a correlation was found between the drop and an increasing serum cortisol level. The initial lymphocyte count could be restored at the earliest 4 days after the operation. However, the regeneration of B lymphocytes occurred much faster than that of T lymphocytes. In addition, only an increase in neutrophils was observed on the second day after the operation.

This study was conducted without specifying the average patient age^35^. Consequently, if the postoperative blood sampling had been done on day 4 in the study conducted here, there might have been no difference from the preoperative measurement values. Moreover, patients undergoing eye surgery receive dexamethasone during the operation for prophylaxis of nausea and vomiting. Since there is a correlation between lymphocyte decline and rising cortisol level, this may have led to an inhibition of the decline by disruption of endogenous cortisol production. The fact that an increase in total lymphocytes in young patients was observed despite dexamethasone administration may be related to the fact that dexamethasone treatment inhibits the cortisol axis of old people more significantly and sustainably than that of young patients. A study with 72 patients (aged 20 to 80 years) of different age groups showed overnight a moderately positive correlation of age and serum cortisol level after the administration of 2 mg of dexamethasone as part of a dexamethasone suppression test^36^. Generally, the cortisol level is increased during and after an operation due to the stress response^37^. In a broader sense, it could be assumed that young patients have more lymphocytes and might thus be better protected, compared to old patients, against postoperative infectious complications.

Another study by Valiathan et al. showed that the relative neutrophil count (in %) continuously increased with age, whereas the lymphocyte count (in %) decreased. Especially from the adult (21 to 50 years) to the elderly group (70 to 92 years), the percentage shares of various immune cells changed: monocytes and neutrophil and eosinophil granulocytes increased, whereas lymphocytes decreased^38^.

The study by Valiathan et al. found that the IL-6 and TNF-α concentrations in elderly patients (70 to 92 years) are much higher compared to the younger adult group (21 to 50 years). The study showed an IL-6 increase from the age of 70 years onwards^38^. Similar findings were seen in the study conducted here. The IL-6 concentration of old patients (> 65 years) was higher than that of young patients. A study by Hsu et al. showed the association between age and IL-6 or CRP increase when performing a colon resection in patients suffering from colorectal carcinoma (*N* = 36). 2 to 48 h after the operation, older patients (≥ 63 years) showed higher IL-6 values than younger patients. After the operation, CRP values in old patients were more elevated compared to those of the young group. Compared to the IL-6 values measured before the operation, a general IL-6 increase was seen in the first two hours after the operation. The values reapproached the initial level after 48 h^39^. The relative change of IL-6 and CRP concentrations due to the operation was not influenced by age in the study conducted here. The lack of difference between the concentration changes in old and young patients may be explained by the fact that a weaker surgical stimulus occurred in the study conducted here, which may have had less influence on CRP or IL-6 concentrations. Moreover, the patients in this study were not additionally burdened by a cancerous disease. Besides, this study showed no difference in the IL-6 and CRP values measured on the first day after the operation compared to those recorded before the operation. Low IL-6 values can be explained by small tissue damage or sterile working methods. Changes in CRP level were probably not detectable due to the short development time. Moreover, across the literature IL-6/CRP levels peak early after after surgical insult. It is possible, that the present study’s timing missed the peak response, which represents a limitation of the analysis^40^.

In previously conducted studies, the neutrophil-to-lymphocyte ratio (NLR) was higher in old patients, especially in those over 70 years of age, than in young patients. The NLR is an indicator of acute infections or an inflammation marker and shows how high the risk of a chronic or cancerous disease is. The older the patients were in the study, the higher was the NLR. But this study did not differentiate between before and after surgery. Only healthy patients (*N* = 362) who had attended the hospital for a physical routine examination were selected^21^. The NLR increase may be related to the already described decrease in lymphocytes and increase in neutrophil granulocytes with age and shows that an influence on the adaptive immune response is detected especially with increasing age. Thus, the decrease in specific defense in old age may result in a higher susceptibility to infections or diseases. The results obtained in this research work initially coincided with the literature. However, no significance was achieved. This may be explained by the small number of cases and the wide scatter of results. Numerically, the NLR was higher in old patients than in young patients, which was, above all, due to the high median values on the day after the operation (post/old). This may explain why older patients are more prone to postoperative complications. However, the NLR of old patients was not conspicuously increased, which could additionally exclude chronic diseases and confirms the positive preselection of patients. Another study had examined 132 patients (> 65 years) undergoing hip surgery. Here, the NLR was recorded before and on the fifth day after the operation. Both the patients who died as a result of the operation and the surviving patients had a similar preoperative NLR, which increased after the operation. On the fifth day after the operation, deceased patients had shown a significantly higher NLR than discharged patients. This gives an indication of the relationship between NLR and increased morbidity^22^.

### Flow cytometric examinations of lymphocytes and granulocytes

#### Antigen expressions CD11b, CD62L and CD66b and TSPO

Neutrophils have different surface receptors whose binding behavior changes during inflammatory processes. CD11b and CD62L antigens are substantially responsible for the adhesion process of granulocytes to the endothelial wall. Mann and Chung (2006) demonstrated that the leukocytes of asthma and COPD patients showed an increase in CD11b expression and CD66b expression and a decrease in CD62L expression. However, this was observed after stimulation with fMLP. When using unstimulated blood, only an increase in CD11b expression in patients with steroid-dependent asthma was observed. When comparing healthy patients to patients with mild and moderate asthma, there was no difference in CD11b expression^41,42^. In the study conducted here, no increase in CD11b expression was found both in old and young patients. This could be an additional proof that no chronically ill persons were included in the study.

A study by Sauce, Dong et al. examined 20 old (70 to 91 years) and 20 young (23 to 35 years) patients. Here, resting PMNs (4 °C) showed no difference in the expression of CD11b and CD62L. After incubation of the cells with PBS (45 min, 37 °C), an increase in CD11b expression and a decrease in CD62L expression was observed in old patients^43^. Contrary to the previously cited literature, the results obtained here showed no significant age- and surgery-related difference in the fluorescence intensity of CD11b, CD62L, CD66b and TSPO antigens of old and young patients. The PMNs in this study were incubated in the dark at 4 °C for 15 min. The study by Sauce, Dong et al. had also shown no differences at 4 °C. There is also a limitation due to the smaller case number, which could have amplified the visible tendency towards an increase or decrease in the measured values. Instead of 20 young and 20 old patients, this study examined only 10 young and 18 old patients.

Sbrana et al. examined the granulocytic CD11b expression in patients (*N* = 20, 43 to 85 years) before, during and after a coronary bypass operation. 5 h after the operation, an increase in CD11b expression was observed in peripheral blood. In the further course, there is a decrease in CD11b surface expression after 24 h. However, statistical significance was observed only in comparison with preoperative blood sampling, which was performed from a coronary vein immediately before cardioplegia^30^. In the study conducted here, both old and young patients did not show any statistically significant differences in CD11b expression from before to after the operation. Possible differences can be explained by the fact that the preoperative blood sample was taken immediately before anesthetic induction and not during the operation performed. Moreover, the postoperative blood sample was always taken on the day after the operation, which is why an adjustment to the preoperative initial value may already have occurred here. Overall, our study may confirm an unchanged ability for PMN adhesion (CD11b as responsible Integrin for PMN adhesion remains unchanged in old age).

In order to be able to examine TSPO in FACS, the outer PMN cell membrane had to be perforated with ethanol before the measurement so that the anti-TSPO antibodies could bind directly to the mitochondria. The subsequent measurements showed mean values of Median TSPO fluorescence intensities to be qualitatively higher in young patients than in old patients. However, no statistically significant difference in TSPO intensities of young and old patients. Despite non reaching significance, it could be assumed that young patients may have either more mitochondria or more TSPO proteins on their mitochondria.

#### Age dependency of CD4+/CD8+ ratio

In the tests performed, old patients showed a higher CD4+/CD8 + ratio than younger patients. Consequently, old patients have less cytotoxic T cells than young patients and/or a larger proportion of T helper cells. The same results could be confirmed by existing literature. In lymphocytes and T cells, respectively, the CD4+/CD8+ ratio changes depending on age and gender^44^. With increasing age, T cell function also changes. A study by Valiathan et al. divided the study patients into five age groups, from infants to the elderly. The age range for adults was set at 21 to 50 years and the age range for elderly patients at 70 to 92 years. Few changes were found when comparing the infants and children groups. Up to adulthood, there was a drop in CD4+ cells (in %) and then, from advanced age, an increase in CD4+ cells. Exactly the opposite was observed for the number of CD8+ cells (in %)^38^. A study by Albertsmeier et al. examined 14 patients undergoing major abdominal surgery. The blood sampling times were 1 h before and 2 h and 24 h after the operation. Here, the CD4+/CD8 + ratio was reduced only slightly. However, with *p* > 0.05, this result was not significant^45^. Comparable findings were seen in the study conducted here with much minor tissue trauma. The comparison of the values measured before and after the operation showed no difference in the CD3+ and CD4+/CD8+ ratio of T cells.

The CD28 expression on T cells is an indicator of aging. The CD8+CD28+ marker labels the cytotoxically active T cells. CD28 expression (CD28+) decreases with age. In patients over 80 years of age, 50 to 60% of the CD8+ T cells showed a decrease in CD28 expression, whereas at birth almost all cells show CD28 expression. Consequently, a percentage increase in CD8+ CD28- and a percentage decrease in CD8+ CD28+ cells are observed with increasing age^46,47^. In this study, the CD8+ lymphocytes of young patients also showed higher CD28+ expression than those of old patients.

#### Perioperative outcoume

Unfortunately, we were unable to establish any correlation between measurements such as NLR or CD4^+^/CD8^+^ ratio and patient outcomes in our study. Nevertheless, in our patient cohort, no postoperative complications were observed, likely reflecting that these were low-risk operative procedures. However, the elevated NLR and altered CD4/CD8 ratios in older patients clearly indicate that older patients are at markedly higher risk than younger patients, and consequently are more prone to developing complications.

While NLR is a marker of inflammation and oxidative stress., the CD4^+^/CD8^+^ ratio indicates the functional balance between T helper cells and cytotoxic T cells, which is the important index concerning internal environment of human immune system. If the ratio decreases, there would be decrement of the immune function in the body^48^.

So far, even in literature there is no robust correlation between the CD4^+^/CD8^+^ ratio and perioperative outcomes or complications. However, there are reports that this ratio exhibited potential independent predictive ability for 60-day functional outcomes of patients with critical intracranial hemorrhage^48^. Nevertheless, several reports indicating the NLR to be a prognostic factor for predicting postoperative patients´ outcome. An increased PMN count and a NLR ≥ 3.5 in the blood of patients are immediate risk factors for the development of postoperative complications like perioperative delirium^49^.

#### Oxidative burst

The concentration of fMLP used in our study did not elicit a substantial effect when applied alone; consequently, TNF-α (TNF) was co-administered to elicit the intended response. This combination (TNF/fMLP) has been previously established and validated in our protocol^50,51^.

The previous literature showed that the fMLP-dependent activation of NAPH oxidase was reduced in old patients. ROS production and thus the ability to kill pathogens was thus negatively influenced by aging^6,52^. Other studies showed that after the activation of PMNs of old patients with TNF-α and subsequent fMLP stimulation or after stimulation with PMA, ROS production was reduced compared to young patients. Here, 20 young and old patients were compared. However, hydroethidine (HE) and whole blood were used here for the measurement of oxygen radicals in the flow cytometer^43^. Another study by Wenisch, Patruta et al. 2000 divided the subjects into three age groups (21 to 36 years, 38 to 56 years, 62 to 83 years). After FITC-labeled Escherichia coli stimulation, both extracellular and intracellular oxidative burst was similar in all age groups. After Staphylococcus aureus stimulation, a decline in ROS production capacity was observed in old patients. This study also used whole blood, as well as dihydrorhodamine (DHR) for visualization, just as the study conducted here^53^. Contrary to the literature, neither young nor old patients showed any significant changes in median fluorescence intensities from before to after the operation after stimulation with PMN, fMLP and TNF-α. Thus, no change in oxidative burst due to the operation was observed both in young and old patients. The differences of the result compared to the existing literature may be explained by the fact that there were differences in the test procedures. Previously performed FACS tests always used whole blood whereas here, granulocytes that were already isolated by means of density gradient centrifugation were used. Stimulation was not performed by means of fluorescence-labeled bacteria, but by means of fMLP. Furthermore, it must be added that in this study there is a prevalence of a sterile inflammation due to the operation, and not a (bacterial) infection, which may result in a more differentiated granulocyte reaction in old age to reduce the cell damage that may occur.

### Live cell imaging of granulocytes of young and old patients

#### Age-related change in migration lengths

The migration distances and the directional accuracy (straightness) of old patients are longer than those of young patients. No statistically significant difference in the track length covered by PMNs due to the operation and the choice of reservoir materials inside the slide was detected. Especially in the first 40 min of the microscope runtime, the migration distances of young and old patients differed clearly. A study by Jayanthi Repalli (2014) showed that cells traveled longer distances in old age. Thus, migration changes with age and becomes less effective as a result (35). Other studies also confirmed that the cells of old patients showed reduced chemotaxis^6,53–55^ towards fMLP. The chemotaxis of granulocytes deteriorated, but the adhesion capacity remained the same in old age^6^. Sapey, Greenwood et al. also described a less directional migration of cells of old patients (> 65 years) in comparison to that of young patients (< 35 years) under attraction with fMLP^54^.

Occasionally, the literature showed no change in migration with age. For example, a study with 25 old (> 65 years) and 25 young patients showed a similar migration and adhesion of old and young PMNs^55^. Another study additionally examined the influence of physical exercise on migratory capacity in old age. The granulocytes of old, athletically active patients (67 ± 5 years) showed a migratory capacity that can be compared to that of the young control group (23 ± 4 years). Compared to that, the migratory capacity of athletically inactive older patients was reduced^56,57^. Thus, the amount of physical exercise of the patients may also have had an influence on the evaluation of the results, but was not recorded in this study.

#### Differences in NETosis of young and old patients

Our experiments showed no statistical differences of ET_50_NETosis between young and old patients, and no differences from before to after the operation. Consequently, we saw no influence of age, surgery or anesthesia on the time of NET formation.

According to the existing literature, ROS production and the capacity for NET formation change with age and are thus reduced^58^. An in vivo experiment with mice showed a lower NET count in older mice than in young mice after 6 h. The same was observed after in vitro stimulation with LPS and TLR2. It was also observed that the PMNs of old mice went earlier into apoptosis than into NETosis^59^. Another study with human granulocytes found that old patients (> 65 years) form more NETs than adult patients (20 to 50 years) after 4 h of LPS stimulation. The percentage of DNA ejected from PMNs of old patients was much higher than that of young adults. However, the NETs of old patients showed less antimicrobial activity against Staphylococcus aureus^60^. Generally, it should be noted that the study conducted here only describes the time course of NET formation and not the extent, number or effectiveness of NETs. If more NETs were formed in young mice than in old mice after 6 h, it can be assumed that NETosis starts later in old mice and thus fewer NETs could be measured. According to Ortmann et al., comparable studies suggest that human granulocytes may also show reduced or attenuated NET formation in old age. This is presumably due to a dysregulation of neutrophil activity and has not been adequately researched yet^61^. Nevertheless, this may argue for an ET_50_ value in old age that is comparable to that of young patients, as NET formation can be equally rapid but weaker or different. Other possible differences in the results may be due to the fact that human and rodent granulocytes behave differently. Moreover, the negative findings regarding NETosis and MPO release could be related to the use of fMLP as a stimulus, which differs from other studies that employ LPS and may drive different inflammatory pathways.

### Ratio of mitochondria to cell count

Our study confirmed that PMNs contain only few mitochondria^62^. Previously, it was already shown that neutrophil granulocytes contain mitochondria and that these show activity^63^. Mitochondria contribute to a major part of neutrophil functions such as NET formation, migration, or adhesion^64^. Our study showed that PMNs of old patients contained more TSPO-labeled mitochondria than those of young patients. With regard to the literature, it would also be possible that more TSPO is present in the mitochondria of old patients and that therefore more “green TSPO areas” or mitochondria are visible. However, in the literature and in previously conducted studies, no human PMNs were used, but various other cell types, such as cerebral cells. A study by Lima, Tamura et al. dealt with neutrophils and TSPO and generally showed higher TSPO expression on circulating neutrophils than in lymphocytes. In addition, an increase in the TSPO level in neutrophils by fMLP stimulation was detectable^65^. Another study with stroke patients determined the plasma TSPO levels at the beginning and end of hospitalization. Here, a correlation between bad clinical outcome and increased plasma TSPO level was seen^66^. Generally, the mitochondrial function decreases with age. Likewise, the volume of mitochondrial DNA is reduced. In addition, mitophagy is inhibited which results in an increase in apoptotic mitochondria in aged tissues^67^. This may be a reason why it appears that older patients have more mitochondria.

At this point it shoud be mentioned, that permeabilization of PMN membranes (e.g. by ethanol) was not necessary in our study, because TSPO could be detected after PMNs have entered NETosis, making the mitochondria freely accessible to the antibody.

### MPO release

In our study no differences of ET_50_MPO nor in young and old patients neither pre or postoperative. In contrast studies to date have shown that MPO plasma levels are increased in old patients, which also has an influence on mortality^57,68^. However, it should be noted that an operation represents a sterile inflammation and not a (bacterial) infection and that it is possible that therefore no enhanced myeloperoxidase release has been seen.

### Change of T_max_ROS in old age

This study showed a later T_max_ROS pre than postoperative. The premature T_max_ROS was more pronounced in younger patients, what is recognizable by means of significant differences (see Fig. 15). In older patients, the prematurity of T_max_ROS caused by surgery was no longer so pronounced, which can be seen in the lack of significance in postoperative T_max_ROS in older patients (see Fig. 15).

### Limitations of the study

For the evaluation and interpretation of the collected data, some systemic limitations of this study must be taken into account:

Despite the exclusion of chronic diseases by anamnesis and review of the patients’ previous diagnostic findings, an undiagnosed or chronic disease cannot be categorically excluded at the time of examination. Moreover, an interference of laboratory chemical examinations with other parameters such as the intake of certain immunomodulating drugs such as ibuprofen or statins should be taken into account. So far, some studies have shown an inflammation-inhibiting effect of statins (3-hydroxy-3-methylglutaryl coenzyme A inhibitors). These influence, for example, endothelial cells and T cells via the Krüppel-like factor (KLF). Thus, leukocyte adhesion and T cell cytotoxicity are reduced^69–71^.

Furthermore, centrifugation was used for the isolation of granulocytes and lymphocytes, which could also have had an influence on cell activity. According to Hundhammer et al., centrifugation can already lead to PMN activation^17,72^.

The collection of patient data was not always the same in all cases. Since the study was conducted in daily clinical practice, it is subject to all the imponderables of daily clinical practice. It cannot be excluded with certainty that, for example, deviating blood sampling times had an influence on the study. In the long term, chronic stress results in a weakening of the immune system. During short-term stress, there is even an increase in the immune response^73^. A study by Liang, Yan et al. 2021 examined two patient groups who underwent laparoscopic cholecystectomy (LC). The intervention group (*n* = 97) should fast for only 6 h before the operation and stop drinking water 2 h before the operation. The control group (*n* = 82) should stop eating food already 12 h before the operation and stop drinking water 6 h before the operation. The patients of the intervention group showed higher preoperative well-being and lower self-rating on the preoperative anxiety scale^74^. Thus, it can be assumed that a prolonged fasting period before the surgical intervention may also result in increased stress, which may also have had an influence on the study conducted here.

No screening for viral cell infection was performed. CD28 downregulation can reflect immunosenescence as well as the infectious burden from chronic viral infections (e.g., CMV, EBV, HIV), which may confound interpretation of CD28 expression in CD8 + T cells across age groups^47^. Moreover, further subdivision of CD8 + T cells based on the expression of CD28 and CD57(both important in the ageing process), would contribute to a more profound comprehension of immunosenescence^75^.

Moreover, the cell counts of the blood cells are subject to different circadian rhythms, which may also have had an effect on the cell counts measured in this study due to the time of day when the blood samples were taken. Leukocyte counts, for example, are higher in the late afternoon and evening than in the morning^76,77^. Akbulut et al. showed that the peripheral blood cell count was lowest during the early morning hours from midnight to 8 am. This was particularly evident in PMNs^77^. According to Swoyer, age has no influence on this circadian rhythmicity of leukocytes and red blood cells. However, the acrophase (maximum) of the number of circulating PMNs and lymphocytes occurs earlier in the elderly (71 ± 5 years) than in younger patients (24 ± 10 years), despite a comparable time of getting up and retiring^78^. This may also have had an influence on the study conducted here due to the two different age groups.

## Conclusion

The effectiveness of the human immune system decreases with increasing age. This effect is known as immunosenescence^5^. In this study, we demonstrated that age might affect PMN and T cell function more than a moderate surgical trauma in combination with general anaesthesia. The results help us to a better understanding of immunosenescence.

## Supplementary Information

Below is the link to the electronic supplementary material.

Supplementary Material 1

Supplementary Material 2

Supplementary Material 3

Supplementary Material 4

Supplementary Material 5

Supplementary Material 6

Supplementary Material 7

Supplementary Material 8

## Data Availability

Our data is available on reasonable request from the authors due to privacy and ethical restrictions. In this case please contact the corresponding author (Richard-Felix.Kraus@klinik.uni-regensburg.de).

## Abbreviations

AB: Antibody
APC: Antigen presenting cell
CD: Cluster of differentiation
CD11b: Integrin alpha M
CD63: Lysosome integral membrane protein1
CXCR: Chemokin receptor
DAMP: Damage associated molecular pattern
FACS: Fluorescence activated cell screening
FSC: Forward scatter
HSC: Hematopoetic stem cell
ICAM-2: Intercellular adhesion molecule 2
Imaris: Interactive microscopy image analysis software
MEM: Minimum essential medium
MPO: Myeloperoxidase
N: Number of experiments
NETs: Neutrophil extracellular traps
PAMP: Pathogen associated molecular pattern
PMA: Phorbol 12-myristate 13-Acetate
PE: Phycoerythrin
Rac 1 und 2: Ras-related C3 botulinum toxin substrate 1 und 2
RT: Room temperature
SD: Standard deviation
SPSS: Statistical package for the social sciences
TCR: T cell receptor
VDAC 1: Voltage-dependent anion-selective channel 1
AFU: Artifical fluorescence units
ATP: Adenosine triphosphate
CD5a: Complement factor C5a
CD62L: L-selektin
CD66b: Carcinoembryonic antigen related cell adhesion molecule 8
CRP: C-reaktive protein
Df: Number of degrees of freedom (Freiheitsgrade)
fMLP: N-Formylmethionyl-Leucyl-Phenylalanin
FITC: Fluorescein-Isothiocyanat
ICAM-1: Intercellular adhesion molecule 1
IL: Interleukine
LPS: Lipopolysaccharide
MHC: Haupthistokompatibilitätskomplex
MV: Mean value
NADP(H): Nicotinamidadenindinucleoidephosphat (protonated)
NK: Natural killer cells
PI: Propidium iodide
PMN: Polymorphkernige neutrophile granulozyten
PBS: Phosphate buffered saline
RPMI: Roswell park memorial institute (medium)
ROS: Reactive oxygen species
SEM: Standard error of the mean
SSC: Side scatter
TNFα: Tumor nekrose factor alpha
