# Supplementary material for: Age affects the immune system more than a moderate surgical trauma and anesthesia
Source: Sci Rep. 2025 Nov 7;15:38993. doi: 10.1038/s41598-025-26401-6 (PMC12595047; doi:10.1038/s41598-025-26401-6)
Supplement: Supplementary file 3 — Supplementary Material 3 [file 41598_2025_26401_MOESM3_ESM.docx]

Table S4: Results of Dunn-Bonferroni-Testing of the NLR

| Groups | Significance | Adjusted significance |
| --- | --- | --- |
| Young_pre_/Young_post_ | 0.84 | 1.0 |
| Young_pre_/Old_pre_ | 0.44 | 1.0 |
| Young_pre_/Old_post_ | 0.02 | 0.09 |
| Young_post_/Old_pre_ | 0.60 | 1.0 |
| Young_post_/Old_post_ | 0.03 | 0.20 |
| Old_pre_/Old_post_ | 0.07 | 0.40 |
