# Supplementary material for: Age affects the immune system more than a moderate surgical trauma and anesthesia
Source: Sci Rep. 2025 Nov 7;15:38993. doi: 10.1038/s41598-025-26401-6 (PMC12595047; doi:10.1038/s41598-025-26401-6)
Supplement: Supplementary file 4 — Supplementary Material 4 [file 41598_2025_26401_MOESM4_ESM.docx]

Table S3: Results of subgroup analysis of leukocyte populations

| **Subgroup** | **Age group** | **Number of measurements (N)** | **Change from pre to post**  **Mean ± SD [%]** |
| --- | --- | --- | --- |
| **Monocytes** | old | 14 | 55.35 ± 54.05 |
| **Monocytes** | young | 9 | 58.75 ± 37.07 |
| **Neutrophils** | old | 18 | 65.26 ± 89.59 |
| **Neutrophils** | young | 9 | 71.54 ± 57.78 |
| **Lymphocytes** | old | 14 | -0.55 ± 32.3 |
| **Lymphocytes** | young | 9 | 48.72 ± 39.80 |
| **Basophils** | old | 18 | -35.24 ± 5.42 |
| **Basophils** | young | 9 | -29.15 ± 25.55 |
| **Eosinophils** | old | 14 | -24.72 ± 109.08 |
| **Eosinophils** | young | 9 | -13.21 ± 52.23 |
