# Supplementary material for: Age affects the immune system more than a moderate surgical trauma and anesthesia
Source: Sci Rep. 2025 Nov 7;15:38993. doi: 10.1038/s41598-025-26401-6 (PMC12595047; doi:10.1038/s41598-025-26401-6)
Supplement: Supplementary file 5 — Supplementary Material 5 [file 41598_2025_26401_MOESM5_ESM.docx]

Table S6: Mean and median AFU of DHR for fMLP and TNFα and PMA stimulation

| **Stimulation** |  | **fMLP + TNFα** | |  |  | **PMA** |
| --- | --- | --- | --- | --- | --- | --- |
| **Age group** | **n** | **Median [AFU]** | |  | **n** | **Median [AFU]** |
| **old** | 36 | 58.1 | |  | 36 | 1332 |
| **young** | 19 | 74.0 | |  | 19 | 1323 |
| **pre/post** |  |  |  | | |  |
| **pre** | 28 | 57.5 | |  | 28 | 1332 |
| **post** | 27 | 63.7 | |  | 27 | 1323 |
