# Supplementary material for: Age affects the immune system more than a moderate surgical trauma and anesthesia
Source: Sci Rep. 2025 Nov 7;15:38993. doi: 10.1038/s41598-025-26401-6 (PMC12595047; doi:10.1038/s41598-025-26401-6)
Supplement: Supplementary file 6 — Supplementary Material 6 [file 41598_2025_26401_MOESM6_ESM.docx]

Table S5: Comparison of the mean values and standard deviations of the pre and post

| Antigen | Age group | n | Pre  Mean ± SD [AFU] | Post  Mean ± SD [AFU] |
| --- | --- | --- | --- | --- |
| CD11b | old | 15 | 435 ± 269 | 385 ± 208 |
| CD11b | young | 7 | 344 ± 141 | 390 ± 223 |
| CD62L | old | 17 | 123 ± 39.8 | 115 ± 38.3 |
| CD62L | young | 7 | 146 ± 25.3 | 118 ± 22.8 |
| CD66b | old | 16 | 267 ± 110 | 142 ± 81.2 |
| CD66b | young | 7 | 206 ± 70.7 | 208 ± 92.4 |
| TSPO | old | 14 | 98.3 ± 93.8 | 95.6 ± 56.6 |
| TSPO | young | 8 | 198 ± 148 | 187 ± 84.4 |
