# Supplementary material for: Age affects the immune system more than a moderate surgical trauma and anesthesia
Source: Sci Rep. 2025 Nov 7;15:38993. doi: 10.1038/s41598-025-26401-6 (PMC12595047; doi:10.1038/s41598-025-26401-6)
Supplement: Supplementary file 7 — Supplementary Material 7 [file 41598_2025_26401_MOESM7_ESM.docx]

Table S7: Overview of the proportion of TmaxROS that could be determined in channels by fluorescence microscopy

| n (analyzable channels)/  n (determinable T_max_ROS) | Young | Old |
| --- | --- | --- |
| prae | 30/10 | 54/10 |
| post | 27/10 | 54/15 |
