# Supplementary material for: Age affects the immune system more than a moderate surgical trauma and anesthesia
Source: Sci Rep. 2025 Nov 7;15:38993. doi: 10.1038/s41598-025-26401-6 (PMC12595047; doi:10.1038/s41598-025-26401-6)
Supplement: Supplementary file 8 — Supplementary Material 8 [file 41598_2025_26401_MOESM8_ESM.docx]

Table S8:Medians and Interquartilranges [min] of ET_50_MPO and ET_50_NETosis

|  | Preoperative [min] | Postoperative [min] |
| --- | --- | --- |
| Median_young_(ET_50_MPO) | 574.2 [IQR 93.4] | 586.7 [IQR 350.6] |
| Median_old_(ET_50_MPO) | 544.4 [IQR 152.3] | 551.9 [IQR 240.2] |
| Median_young_(ET_50_NETosis) | 586.5 [IQR 260.0] | 559.1 [IQR 366.3] |
| Median_old_(ET_50_NETosis) | 582.0 [IQR 296.2] | 498.1 [IQR 274.6] |
